# Acute Augmentations to Psychological Therapies in Eating Disorders: A Systematic Review and Meta-Analysis

**DOI:** 10.1007/s11920-024-01519-y

**Published:** 2024-08-02

**Authors:** Jamie-Lee Pennesi, Catherine Johnson, Marcela Radünz, Tracey D. Wade

**Affiliations:** Flinders University Institute for Mental Health and Wellbeing and Blackbird Initiative, Flinders University, Adelaide, SA Australia

**Keywords:** Acute augmentation, Eating disorders, Therapy, Meta-analysis, Systematic review

## Abstract

**Purpose of Review:**

The purpose of this systematic review and meta-analysis was to examine the use and efficacy of acute augmentation therapies in eating disorders.

**Recent Findings:**

A meta-analysis addressing this topic across psychological disorders found augmentation significantly improved therapy outcome with strongest findings for augmentations targeting biological mechanisms; however, only one study examined eating disorders.

**Summary:**

Our systematic review identified 29 studies examining people with eating disorders (*N* = 1831 participants, 93.7% female), of which 17 RCTs (*n* = 1162 participants) were included in the meta-analysis. Small subgroups of acute augmentations were identified. Adding acute augmentations to an intervention was effective in 72.4% of studies, with a significant effect on eating disorder outcomes (Hedges’ *g* = 0.14, 95% CI: [0.02, 0.26]). Acute augmentation looks to be a promising approach regardless of weight status or whether it is added to treatment as usual or a single therapy approach.

**Supplementary Information:**

The online version contains supplementary material available at 10.1007/s11920-024-01519-y.

## Introduction

Eating disorders represent a complex and varied range of disorders, across six main eating and feeding disorders. Three of these eating disorders (anorexia nervosa, bulimia nervosa, and binge eating disorder) and the residual category of Other Specified Feeding and Eating Disorders (OSFED), are associated with evidence-based treatment. OSFED does not differ from the other eating disorders in terms of risk factors or impairment [1–3]; even unhealthy weight control behaviours that do not meet diagnosis are associated with significantly higher economic costs among adolescents [4]. These eating disorders are associated with an almost four-fold increased mortality compared to age- and sex-matched populations, the highest of any psychiatric disorder after substance use disorders [5]. Treatment options range from outpatient psychological treatment involving family members in an age-appropriate manner (for mild or moderately severe cases) through to inpatient or day patient treatment for those with more severe medical or psychological risk, required for up to 35% of patients [6].

While large within-group effect sizes are obtained for eating disorder treatments using cognitive behaviour therapy (CBT) in routine clinical care, with an attrition rate of 25.5% [7], CBT for bulimia nervosa produces abstinence in only 37.5% of completers [8]. Specific psychological treatments for adult outpatients with anorexia nervosa are associated with modest improvements but no reliable evidence supports superiority of one approach over specialist, supportive psychotherapy [9]. This situation is mirrored in other mental health problems, where “even the best therapies leave substantial proportions of patients with ongoing clinical problems” [p. 389, 10]. At least half of all patients with mental health disorders do not respond adequately to psychological therapy. For this reason, acute augmentations for psychological therapy have been proposed as a pathway to improve outcomes, especially where there is comorbidity and such augmentation may have transdiagnostic mechanisms and utility [10, 11].

Acute augmentations have been defined as interventions delivered immediately before, during, or after a session of manualised psychological therapy with the aim of enhancing the impact of the therapy [10]. They can be delivered as a one off, but can also be repeated across multiple therapy sessions. We note that acute augmentations are distinct from combination treatments, in which two independent therapies or therapeutic approaches are delivered on a long-term basis in parallel to potentially provide additive benefits (e.g., medication prescribed alongside a course of psychological therapy) [10]. In contrast, acute augmentations target specific biological or psychological processes on at least one occasion to boost outcomes for an evidence-based treatment (e.g., bias modification or imagery might be used to reduce social anxiety such that therapeutic tasks can be better utilized by the patient [12, 13]). Further detailed definitions are provided in Supplementary Information S1.1. Differences between acute augmentations and other approaches, such as combination therapies, are described in Supplementary Information S1.2.

A meta-analysis comparing acute augmentations with a comparison group [10] found a small but significant between group effect size (Hedges’ *g* = −0.27, 95% CI: [−0.36, −0.18]) impact on severity of mental health problems favouring augmentation. Biological augmentation (e.g., medication, brain stimulation) provided the strongest effects, with some promise shown by interventions involving processing memories, imagery, motivational enhancement, and bias modification [10]. Despite there being many new augmentation techniques introduced to reduce eating disorder habits and promote new learning [6], the meta-analysis only included one study that included a population with an eating disorder. This one study, published ten years ago, investigated the use of virtual reality to enhance CBT for people with obesity and binge eating disorder [14]. It appeared that the search terms in the meta-analysis resulted in the omission of key studies; only the terms “eating disorder” and “binge eating” were included in the title search rather than the wider range of eating disorders. The purpose of this review and meta-analysis is to therefore redress this omission and to examine the use and efficacy of acute augmentation therapies in eating disorders, given the potential importance of this approach to improve treatments [6].

## Method

### Search Strategy and Selection Criteria

The present study was conducted and reported in line with the evidence-based guidelines [15, 16] and was preregistered on PROSPERO (CRD42024506142) (registered on 25 January 2024; updated 6 February 2024). Deviations from the protocol are summarised in Supplementary Information S1.3. The databases Medline, Scopus, and PsycINFO were searched for eligible studies for all years covered through to 29 January 2024, limited to English language papers. There were no restrictions placed on the date of publication. ClinicalTrials.gov was searched for unpublished studies. We also performed reference tracing for additional studies meeting inclusion criteria. The full search terms used are provided in Supplementary Information S1.4. We used a similar search strategy to Nord et al. [10], combining psychological therapy terms with eating disorder-related terms, augmentation-related terms, and trial-related terms, using Boolean operators and database Term Finders as available. We expanded on the terms included in Nord et al. [10] to include a wider range of eating disorders and behaviours and added terms to better capture psychological therapies and designs commonly used in eating disorders (e.g., family-based treatment, stepped care). We removed terms not related to eating disorders. Additional search terms were identified by looking at words in the titles and abstracts of six known relevant studies.

Studies that met the following criteria were included: (1) randomised controlled trials, non-randomised controlled trials, pilot studies, feasibility trials, or unpublished studies; studies that included (2) at least one session of manualised psychological therapy for eating disorders, which could include evidence-informed treatment-as-usual (TAU), and at least one acute augmentation aimed at enhancing the therapeutic impact of the psychological therapy; (3) a control or comparison group; (4) at least one validated continuous psychological outcome assessed before and after treatment; (5) participants with a diagnosed or probable eating disorder according to a validated questionnaire or interview assessment, unless diagnosis was confirmed via specialist assessment from an inpatient, outpatient, or specialised eating disorder centre; and (6) the study was written in English (Supplementary Information S1.1).

Studies were excluded if they met the following criteria: (1) reviews, meta-analyses, case reports, case series, commentaries, editorials, study protocols, conference abstracts, animal studies, books, or qualitative studies; (2) the study was (i) not focused on eating disorders, unless an eating disorder was co-occurring (e.g., obesity with an eating disorder), (ii) not focused on treatment, (iii) an adaptive or stepped care design; (3) the psychological therapy was (i) not manualised, (ii) entirely self-guided; (4) the acute augmentation was (i) a combination treatment, (ii) an integration of two treatments or treatment approaches; (5) there was no control or comparison group; (6) there was no continuous psychological outcome measure; (7) the study was still being conducted; and (8) the data were insufficient to calculate effect sizes. More information on inclusion and exclusion criteria is provided in Supplementary Information S1.5.

Studies retrieved were downloaded and duplicates were subsequently removed (manually and by Covidence). To determine study suitability, we used a two-step approach managed by Covidence. First, two independent reviewers (JP and CJ) screened study titles and abstracts of at least 20% of identified articles to examine whether they related broadly to the question of interest. Any discrepancies were resolved by discussion. One reviewer (JP) then screened all remaining titles and abstracts. Second, two independent reviewers (JP and MR) examined the full texts of at least 20% of included studies to assess eligibility for inclusion in the systematic review and meta-analysis. Discrepancies were again resolved by discussion. One reviewer (JP) then examined all remaining full texts. The inter-rater reliability measured by Cohen’s Kappa at title and abstract was 0.50 (20.3% of titles and abstracts) and at full text screening was 0.83 (22.6% of full texts), indicating moderate to high inter-rater reliability during the study selection process.

### Data Extraction Process

Two authors (JP and CJ) independently extracted information required for the qualitative synthesis and meta-analysis. The following information was extracted from each eligible study: author, publication year, country, study design, sample size, participant demographics including mean age, gender, eating disorder diagnosis, types of interventions (augmentation and control groups), intervention duration, psychological assessment measures and pre-post assessment period, and continuous outcome data. Specifically, to calculate between-group effect sizes and within-group mean gain scores, the following outcome data were extracted: sample size, means, and standard deviations for augmentation and control groups at baseline and post-treatment. If a study contained more than one potential comparator, we selected the group that represented the control group/true comparator (i.e., psychological therapy but no augmentation or active intervention). Studies that did not include a control group/true comparator were excluded.

### Statistical Analysis

#### Efficacy of Acute Augmentation

Within-group mean gain scores were calculated for augmentation and control groups in each study in Excel using the formula: baseline mean – end of treatment mean, where higher scores indicated greater improvement in symptoms. This was used as a proxy for intervention efficacy within each study; that is, how much symptoms improved from baseline to end of treatment. Pre-calculated mean gain (improvement) scores for augmentation and control groups in each study were then compared to determine whether augmentation was more effective (i.e., showed a greater improvement in symptoms) than the control and, specifically, whether augmentation made the psychological therapy more effective than no augmentation.

#### Meta-Analyses

First, a meta-analysis of randomised controlled trials (RCTs) examining the efficacy of acute augmentation to psychological therapy on eating disorder outcomes was conducted. A decision was made to include only RCTs and studies with eating disorder outcomes in the meta-analysis to examine the most robust of augmentation studies and to ensure homogeneity across outcomes and maintain the focus on eating disorders. Next, two subgroup analyses were conducted to examine the efficacy of acute augmentation by weight status (underweight versus non-underweight) and by therapy type (TAU versus single therapy approaches such as CBT). Meta-analyses were conducted using the *R* statistical software program using the *meta* package [17]. Between-group effect sizes were pre-calculated using the online Campbell Collaboration tool (https://campbellcollaboration.org/research-resources/effect-sizecalculator.html), inputting mean and standard deviation gains between baseline and end of treatment for augmentation and control groups plus sample size at baseline. As no studies included in the analysis reported *r* coefficient for pre-post for each group, the Campbell Collaboration tool assigns a default* r* coefficient of 0.5. The pre-calculated Hedges’ *g* effect sizes were then pooled using random effect meta-analyses investigating the overall effect of the acute augmentation on eating disorder outcomes. Forest plots were produced using Hedges’ *g* values and 95% confidence intervals. Hedges’ *g* is recommended for use with small sample sizes and when comparison groups have varying sample sizes [18, 19], with recommended interpretation as 0.2 = small, 0.5 = medium, and 0.8 = large [20].

Subgroup analyses were conducted using a mixed effects model in which the effect sizes within the subgroups are pooled with a random effects model and tested as to whether they differ between the subgroups using a fixed effect model [21].

#### Heterogeneity

Heterogeneity denotes whether the variability in effect sizes across studies is greater than what would be expected due to random error alone [22]. The heterogeneity of the data was evaluated using *Q* and *I*^*2*^ statistics. The *Q* statistic is a measure of weighted squared deviation around the weighted mean effect size, with a significant result suggesting that variability is unlikely to be due to chance [23]. The *I*^*2*^ statistic is a measure of the proportion of total study variation that is due to heterogeneity. A value of zero indicates no variance between study estimates is due to heterogeneity, values of 30 or less indicate mild heterogeneity, and values above 50 indicate notable heterogeneity [24].

#### Publication Bias

Egger’s regression intercept was utilised to evaluate the presence of publication bias [25]. This method involves examining the correlation between effect sizes and standard errors of effect sizes to determine if there is a significant association between study effect size and study precision. A regression intercept of zero is expected if there is no publication bias, with significant results suggesting presence of publication bias.

## Results

### Initial Searches

**Fig. 1:**
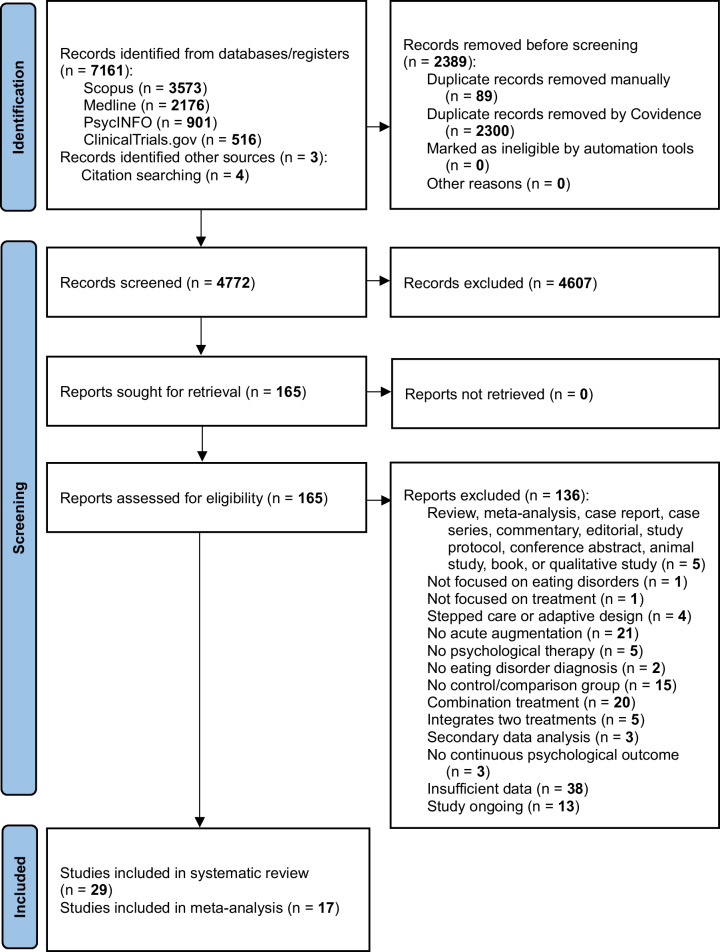
PRISMA flow diagram for systematic review

The initial search (conducted 29 January 2024) identified 7157 studies. A further four studies were identified from other sources (Fig. 1). After removal of duplicates, 4772 studies remained. Two reviewers (JP and CJ) screened 20.3% of titles and abstracts and one reviewer (JP) screened the remaining titles and abstracts, excluding 4607 studies and leaving 165 studies remaining. Two reviewers (JP and MR) then screened 22.6% full texts and at least one reviewer (JP or MR) screened the remaining full texts, excluding a further 136 studies. Supplementary Information S1.6. provides a summary of excluded studies with reasons. Key reasons for exclusion were insufficient data (*n* = 38; 27.9% of excluded), no acute augmentation (*n* = 21; 15.4%), and augmentation was a combination treatment (*n* = 20; 14.7%). Any discrepancies were resolved by discussion. A final 29 studies met inclusion criteria and were included in the systematic review [26–54]. Of these, 17 RCTs that had eating disorder outcomes were included in the meta-analysis [26, 28–31, 35, 37, 40–44, 48, 49, 51–53].

### Systematic Review

#### Characteristics of the Included Studies

The final sample for the systematic review consisted of 1831 individuals with a mean age of 29.31 years (*SD* = 10.62; female = 93.7%) (Table 1). Research was most frequently conducted in the United States (*n* = 6; 20.7%) but with wide representation including Australia (*n* = 5; 17.2%), the United Kingdom (*n* = 4; 13.8%), Italy (*n* = 3; 10.3%), Canada (*n* = 2; 6.9%), the Netherlands (*n* = 2; 6.9%), Spain (*n* = 2; 6.9%), Austria (*n* = 1; 3.4%), France (*n* = 1; 3.4%), Israel (*n* = 1; 3.4%), and Japan (*n* = 1; 3.4%). Sixteen (55.2%) studies contained mixed eating disorder samples, while the remainder (*n* = 13, 44.8%) contained participants with just one eating disorder diagnosis (e.g., anorexia nervosa, binge eating disorder).

**Table 1:** Characteristics of studies included in the systematic review

**Authors (year of publication) (country)**	**Included *N*** **(1) Augmentation &** **(2) Control group**	**Mean (SD) age** **(1) Augmentation &** **(2) Control group**	**Gender (% female)**	**ED Dx (%)**	**Weight status**	**Psychological ED measure**	**Interventions** **(1) Augmentation &** **(2) Control group**	**Type of augmentation**	**Augmentation efficacy**	**Duration**
Agras et al. (1989) [26]^a, b^ (United States)	(1) 16 (2) 17	29.2 (8.6)^c^	100	BN = 100	Non-underweight	EAT DIET + EDI DT^d^	(1) CBT + ERP (2) CBT	ERP-based	Augmentation < Control	4 months
Allen et al. (2012) [27] (Australia)	(1) 52 (2) 43	(1) 26.52 (8.98) (2) 26.44 (8.98)	NR	AN = 22.1; BN = 32.6; EDNOS “AN-like” = 35.8; EDNOS “BN-like” = 6.3; EDNOS-P = 2.1; UFED = 1.1	Non-underweight	—	(1) TAU + MFT (2) TAU	Motivation-focused	Augmentation > Control	NR (range of Tx = 10–100 sessions)
Anastasiadou et al. (2020) [28]^a^ (Spain)	(1) 53 (2) 53	(1) 17.25 (3.54) (2) 18.88 (7.77)	91.4	AN-R = 48.1, AN-P = 6.6; BN = 12.3, BED = 3.8, OSFED = 29.2	Underweight	EDE-Q global	(1) TAU + TCApp (2) TAU	App-based	Augmentation > Control	12 weeks
Boerhout et al. (2017) [29]^a^ (Netherlands)	(1) 38 (2) 32	(1) 25.00 (7.59) (2) 23.34 (7.30)	94.3	AN = 55.7; BN = 32.9; BED = 4.3; OSFED = 7.1	Underweight	EDE-Q global	(1) TAU + body and movement-oriented intervention (2) TAU	Movement/exercise-based	Augmentation > Control	6 weeks
Cardi et al. (2019) [30]^a^ (United Kingdom)	(1) 99 (2) 88	(1) 26.60 (8.46) (2) 29.15 (10.03)	96.8	AN or AAN (breakdown NR)	Underweight	EDE-Q global	(1) TAU + RecoveryMANTRA (2) TAU	Motivation-focused	Augmentation > Control	6 weeks
Cesa et al. (2013) [31]^a^ (Italy)	(1) 27 (2) 20	(1) 32.90 (8.80) (2) 29.9 (7.95)	100	BED = 100	Non-underweight	BIAQ	(1) CBT + VR-enhanced CBT (2) CBT	CBT-based	Augmentation > Control	6 weeks
Children's Hospital of Philadelphia (2019) [32]^b, e, f^ (United States)	(1) 19 (2) 21	(1) 15.25 (1.61) (2) 15.51 (1.81)	62.1	AN = 100	Underweight	—	(1) FBT + adolescent-focused CRT (2) FBT	CRT-based	Augmentation < Control	6 months
	(1) 19 (2) 21	(1) 15.21 (1.53) (2) 15.51 (1.81)	62.1	AN = 100	Underweight	—	(1) FBT + parent-focused CRT (2) FBT	CRT-based	Augmentation < Control	6 months
Compare et al. (2016) [33] (Italy)	(1) 63 (2) 55	(1) 51.10 (4.10) (2) 50.80 (6.00)	57.6	BED = 100	Non-underweight	—	(1) EFGT + dietary counselling (2) EFGT	Other	Augmentation > Control	20 weeks
Dean et al. (2009) [34] (Australia)	(1) 19 (2) 16	(1) 21.30 (7.90) (2) 23.84 (6.60)	100	AN-R = 35.7; AN-BP = 14.3; BN = 2.4; EDNOS BN = 40.5; EDNOS AN = 7.14	Underweight	—	(1) TAU + group MET (2) TAU	Motivation-focused	Augmentation > Control	4 weeks
Dingemans et al. (2013) [35]^a^ (Netherlands)	(1) 41 (2) 41	(1) 27.00 (8.60) (2) 26.90 (8.00)	100	AN-R = 46.3; AN-BP = 25.6; BN = 11.0; EDNOS AN-R = 9.8; EDNOS AN-BP = 7.3	Underweight	EDE-Q global	(1) TAU + CRT (2) TAU	CRT-based	Augmentation > Control	6 weeks
Fernandez-Aranda et al. (2015) [36] (Spain)	(1) 20 (2) 18	29.50 (9.90)^c^	100	BN = 100	Non-underweight	—	(1) TAU + SVG (2) TAU	Other	Augmentation > Control	16 weeks
Flynn et al. (2023) [37]^a, b^ (United Kingdom)	(1) 20 (2) 20	(1) 40.40 (9.83) (2) 43.70 (10.60)	97.6	BED = 100	Non-underweight	EDE-Q global	(1) ABM Training + real tDCS (2) ABM Training	Neurological	Augmentation > Control	NR (2–3 weeks of Tx)
Galasso et al. (2020) [38] (Italy)	(1) 10 (2) 9	(1) 54.00 (11.00) (2) 53.00 (13.00)	100	BN = 100	Non-underweight	—	(1) TAU + CAAET (2) TAU	Movement/exercise-based	Augmentation > Control	6 months
Ganci et al. (2018) [39] (Australia)	(1) 40 (2) 40	(1) 15.20 (1.70) (2) 15.20 (1.50)	80.0	AN or ARFID (breakdown NR)	Underweight	—	(1) FBT + parent education and skills workshop (2) FBT	Family-based	Augmentation > Control	NR (> 12 weeks)
Golan (2022) [40]^a^ (Israel)	(1) 47 (2) 47	(1) 24.40 (0.86) (2) 24.20 (3.10)	100	AN = 37.0; BN = 41.0; BED = 11.0; ARFID =< 10.0	Non-underweight	EDE-Q global	(1) MBT + ECOSA (2) MBT	Other	Augmentation = Control	8 months
Goldstein et al. (2014) [41]^a^ (Australia)	(1) 28 (2) 29	(1) 23.43 (7.51) (2) 22.97 (6.72)	98.4	AN-R = 24.6; AN-BP = 15.8; BN = 24.6; EDNOS-R = 10.5; EDNOS-BP = 24.6	Non-underweight	EDE-Q global	(1) TAU + CBT-P (2) TAU	CBT-based	Augmentation < Control	NR (3.5 weeks of Tx)
Grilo et al. (2013) [42]^a^ (United States)	(1) 24 (2) 24	(1) 45.00 (11.80) (2) 46.50 (10.20)	79.2	BED = 100	Non-underweight	EDE-Q global	(1) Usual care + SH-CBT (2) Usual care	CBT-based	Augmentation > Control	4 months
Keeler et al. (2022) [43]^a^ (United Kingdom)	(1) 40 (2) 40	(1) 30.00 (23.00, 40.75)^g^ (2) 29.00 (23.00, 35.00)^g^	96.3	BN = 66.0; BED = 34.0	Non-underweight	EDE-Q global	(1) TAU + FoodT app (2) TAU	App-based	Augmentation > Control	4 weeks
Keshen et al. (2020) [44]^a^ (Canada)	(1) 40 (2) 41	(1) 27.90 (9.11) (2) 27.64 (10.39)	100	AN 18.5; AN-BP 21.0%; BN 38.3; OSFED 8.6; UFED 13.6	Non-underweight	EDE-Q global	(1) TAU + Recovery Record (2) TAU	App-based	Augmentation < Control	NR (up to 32 weeks of Tx)
Lackner et al. (2016) [45] (Austria)	(1) 10 (2) 12	NR (range = 12–18 years)	100	AN = 100	Underweight	—	(1) TAU + EEG neurofeedback training (2) TAU	Neurological	Augmentation > Control	5 weeks
Nakazato et al. (2009) [46] (Japan)	(1) 16 (2) 25	(1) 23.2 (4.2) (2) 23.8 (5.9)	97.6	AN-BP = 14.6; BN-P = 63.4; BN-NP = 19.5; EDNOS = 2.4	Non-underweight	—	(1) CBT + MET (2) CBT	Motivation-focused	Augmentation < Control	15 weeks
Pendleton et al. (2002) [47]^b^ (United States)	(1) 20 (2) 17	45.00 (8.30)^c^	100	BED = 100	Non-underweight	—	(1) CBT + exercise intervention (2) CBT	Movement/exercise-based	Augmentation > Control	4 months
Presseller et al. (2022) [48]^a^ (United States)	(1) 29 (2) 26	(1) 38.07 (13.59) (2) 39.58 (15.04)	83.6	BN spectrum = 100	Non-underweight	EDE global	(1) CBT-E + JITAIs (2) CBT-E	Other	Augmentation < Control	16 weeks
Reyes-Rodriguez et al. (2021) [49]^a^ (United States)	(1) 12 (2) 13	(1) 39.80 (8.40) (2) 35.40 (9.40)	100	BN-NP = 28.0; BN-P = 20.0; BED = 28.0; EDNOS = 24.0	Non-underweight	EDE-Q global	(1) CBT + family enhanced model (2) CBT	Family-based	Augmentation > Control	NR (28 sessions of Tx)
Rigaud et al. (2011) [50] (France)	(1) 52 (2) 51	(1) 27.4 (8.1) (2) 27.9 (6.2)	100	AN = 35.0; BN = 65.0	Non-underweight	—	(1) CBT + tube feeding (2) CBT	Other	Augmentation > Control	2 months
Rowlands et al. (2022) [51]^a^ (United Kingdom)	(1) 37 (2) 30	(1) 16.36 (1.59) (2) 15.47 (1.78)	98.0	AN = 92.0; BN = 4.0; EDNOS = 2.0; ARFID = 2.0	Underweight	EDE-Q global	(1) TAU + online CBMT (2) TAU	Other	Augmentation > Control	Range = 3–24 weeks
Trottier et al. (2015) [52]^a^ (Canada)	(1) 22 (2) 21	24.8 (7.3)^c^	100	AN = 26.7; BN = 64.4; EDNOS = 8.9	Non-underweight	EDE WC	(1) Maintenance TAU + graded body image exposure (2) Maintenance TAU	Other	Augmentation > Control	NR (4–16 weeks of Tx)
Wade et al. (2009) [53]^a^ (Australia)	(1) 22 (2) 25	(1) 24.28 (6.37) (2) 19.71 (3.10)	95.7	AN = 100	Underweight	EDE global	(1) TAU + MI (2) TAU	Motivation-focused	Augmentation > Control	2 weeks
Wilson et al. (1991) [54] (United States)	22^ h^	(1) 19.80 (SD NR) (2) 21.60 (SD NR)	NR	BN = 100	Non-underweight	—	(1) CBT + ERP (2) CBT	ERP-based	Augmentation < Control	20 weeks

Augmentation efficacy refers to whether the augmentation was more (indicated by >) or less (indicated by <) effective than the control, or as effective as the control (indicated by =) from baseline to end of treatment

*N* sample size, *SD* standard deviation, *ED Dx* eating disorder diagnosis, *ED* eating disorder, *NR* not reported, *Tx* treatment

ED Dx: *AAN* atypical anorexia nervosa, *AN* anorexia nervosa, *AN-BP* anorexia nervosa, binge-purge type, *AN-P* anorexia nervosa, purging type, *AN-R* anorexia nervosa, restricting type, *ARFID* avoidant/restrictive food intake disorder, *BED* binge eating disorder, *BN* bulimia nervosa, *BN-NP* bulimia nervosa, non-purging type, *BN-P* bulimia nervosa, purging type, *EDNOS* eating disorder not otherwise specified, *EDNOS AN* eating disorder not otherwise specified, anorexia nervosa, *EDNOS “AN-like” *eating disorder not otherwise specified, anorexia nervosa, *EDNOS AN-BP* eating disorder not otherwise specified, anorexia nervosa, binge-purge type, *EDNOS AN-R* eating disorder not otherwise specified, anorexia nervosa, restricting type, *EDNOS BN* eating disorder not otherwise specified, bulimia nervosa, *EDNOS “BN-like”* eating disorder not otherwise specified, bulimia nervosa, *EDNOS-BP* eating disorder not otherwise specified, binge-purge type, *EDNOS-P* eating disorder not otherwise specified, purging disorder, *EDNOS-R* eating disorder not otherwise specified, restricting type, *OSFED* other specified feeding or eating disorder, *UFED* unspecified feeding or eating disorder. Categories were labelled as described by each study

Psychological ED measures: *BIAQ* Body Image Avoidance Questionnaire, *EAT DIET* Eating Attitudes Test, Dieting subscale, *EDE global* Eating Disorder Examination, global score, *EDE WC* Eating Disorder Examination, Weight Concern subscale, *EDE-Q global* Eating Disorder Examination-Questionnaire, global score, *EDI DT* Eating Disorder Inventory, Drive for Thinness subscale

Psychological therapies, augmentation, and control groups: *ABM* attention bias modification training, *CAAET* combined aerobic and anaerobic exercise training, *CBMT* cognitive bias modification training for social stimuli, *CBT* cognitive behavioural therapy, *CBT-E* enhanced cognitive behavioural therapy, *CBT-P* cognitive behavioural therapy for perfectionism, *CRT* cognitive remediation therapy, *ECOSA* eating and control styles axis, *EEG* electroencephalogram, *EFGT* emotionally focused group therapy, *ERP* exposure with response prevention, *JITAIs* just-in-time adaptive interventions, *Maintenance TAU* maintenance treatment-as-usual, *MBT* mentalisation-based psychotherapy, *MET* motivational enhancement therapy, *MFT* motivation-focused therapy, *MI* motivational interviewing, *RecoveryMANTRA* guided self-help intervention from Maudsley model of anorexia nervosa treatments for adults, *SH-CBT* self-help cognitive behavioural therapy, *SVG* serious video games, *TAU* treatment-as-usual, *tDCS* transcranial direct current stimulation, *VR-enhanced CBT* virtual reality-enhanced cognitive behavioural therapy

Augmentation subgroups: *CBT* cognitive behaviour therapy, *CRT* cognitive remediation therapy, *ERP* exposure with response prevention. Subgroups consist of augmentations used in more than one study. Augmentations used in only one study were included in the ‘Other’ subgroup

^a^ indicates RCTs included in the meta-analysis (*n* = 17)

^b^ study includes additional groups not reported in the present study

^c^ indicates the mean age (SD) for the sample

^d^ indicates the average of the EAT Dieting subscale and the EDI Drive for Thinness subscale

^e^ study included two independent acute augmentation groups compared to control; individuals were only counted once in descriptives

^f^ author represents clinical trial sponsor as reported on ClinicalTrials.gov as does not report author(s)

^g^ indicates the median age (interquartile range) for the sample

^h^ indicates *N* for the sample

Augmentations to TAU were investigated in just over half of the studies (*n* = 16; 55.2%), followed by augmentations to psychological therapies focused on CBT (*n* = 8; 27.6%), family-based treatment (*n* = 2; 6.9%), attention bias modification (*n* = 1; 3.4%), emotionally focused group therapy (*n* = 1; 3.4%), and mentalisation (*n* = 1; 3.4%). Evidence- informed TAU could consist of any of the following: CBT (e.g., individual or group), individual or group psychotherapy, family psychotherapy, outpatient or community care (e.g., visits with primary care physician or other healthcare providers, after care at home, medication management, symptom monitoring), inpatient care, intensive treatment program, day treatment, dietary counselling, behavioural weight gain programme, or input from a multidisciplinary team (e.g., psychiatrist, psychologist, psychomotor therapist, nurse practitioner, nurse, dietician, nutritionist). Half of the 16 studies with TAU (50.0%), reported elements of CBT (i.e., elements of CBT or dialectical behaviour therapy in one study) as part of TAU. We note that TAU reflects common real-world practice [55]. The majority of studies included augmentations that were delivered ‘during’ the psychological therapy (*n* = 27; 93.1%), with only two studies (6.9%) with augmentations delivered ‘before’ therapy.

#### Included Acute Augmentations

A summary of specific acute augmentations included in the systematic review appears in Table 2. Eight subgroups of augmentation could be identified (i.e., augmentations used in more than one study). The most investigated was forms of motivational enhancement (*n* = 5; 17.2%), followed by CBT-based interventions (*n* = 3; 10.3%), App-based interventions (*n* = 3; 10.3%), movement-related interventions (*n* = 3; 10.3%), cognitive remediation therapy (*n* = 2; 6.8%), family-based interventions (*n* = 2; 6.8%), exposure and response prevention (*n* = 2; 6.8%), and neurological interventions (*n* = 2; 6.8%). Other augmentations (*n* = 7; 24.1%) were only included in one study (e.g., dietary counselling, bias modification). The subgroup of augmentation against each study is shown in Table 1.

**Table 2:** Summary of subgroups and acute augmentations included in the systematic review

**Subgroup & acute augmentation**
Motivation-focused interventions
Motivational interviewing (MI)^a^
Motivation-focused therapy (MFT)
Motivational enhancement therapy (MET)
Group-based motivational enhancement therapy (Group MET)
Guided self-help intervention from Maudsley model of anorexia nervosa treatments for adults (RecoveryMANTRA)^a^
Cognitive behaviour therapy-based interventions
Cognitive behaviour therapy for perfectionism (CBT-P)^a^
Self-help cognitive behaviour therapy (SH-CBT)^a^
Virtual reality-enhanced cognitive behaviour therapy (VR-enhanced CBT)^a^
App-based interventions
TCApp^a^
FoodT app^a^
Recovery Record^a^
Movement/exercise-based interventions
Body and movement-oriented intervention^a^
Exercise intervention
Combined aerobic & anaeobic exercise training (CAAET)
Cognitive remediation therapy-based interventions
Cognitive remediation therapy (CRT)^a^
Adolescent-focused cognitive remediation therapy (Adolescent-focused CRT) or Parent-focused cognitive remediation therapy (Parent-focused CRT)
Family-based interventions
Family enhanced model^a^
Parent education and skills workshop
Exposure with response prevention-based interventions
Exposure with response prevention^a^
Exposure with response prevention
Neurological interventions
Transcranial direct current stimulation (tDCS)^a^
EEG neurofeedback training
Other interventions^b^
Dietary counselling
Cognitive bias modification training for social stimuli (Online CBMT)^a^
Graded body image exposure^a^
Serious video games (SVG)
Eating and control styles axis (ECOSA)^a^
Just-in-time adaptive interventions (JITAIs)^a^
Tube feeding

^a^ indicates acute augmentations included in the meta-analysis

^b^ indicates acute augmentations used in only one study

#### Efficacy of Acute Augmentation

A summary of whether augmentation was more or less effective (i.e., showed a greater or lesser improvement in symptoms from baseline to end of treatment) than the control in each study is included in Table 1. Acute augmentation was more effective than the control group in the majority of studies (*n* = 21, 72.4%), but less effective than the control in some studies (*n* = 7, 24.1%), and as effective as the control in one study (3.4%). This suggests that augmentation in addition to the psychological therapy was more effective than the psychological therapy alone (with no augmentation) in most but not all studies.

### Meta-analysis

#### Characteristics of the Included RCTs

A subset of 17 RCTs that provided eating disorder outcomes were included in the meta-analysis. The final sample consisted of 1162 individuals with a mean age of 28.80 years (*SD* = 8.44; female = 96.1%) (Table 1). The majority investigated augmentations to TAU (*n* = 11; 64.7%), and all (100%) included augmentations that were delivered ‘during’ the psychological therapy. Acute augmentation was more effective than the comparison condition in the large majority of studies (*n* = 12, 70.6%).

#### Included Acute Augmentations

Three subgroups of augmentation could be identified, with the most common forms being CBT-based interventions (*n* = 3; 17.6%) and App-based interventions (*n* = 3; 17.6%), followed by motivational enhancement (*n* = 2; 11.8%). The subgroup of augmentation against each study is shown in Table 1.

#### Data Synthesis

A meta-analysis of RCTs was conducted for eating disorder outcomes (*n* = 17), most frequently the the Eating Disorder Examination Questionnaire global score. Results showed a significant effect of acute augmentations with a small effect size (Hedge’s *g* = 0.14; 95% CI: [0.02, 0.26]). Cochrane’s *Q* (*Q* = 14.76, *p* = .54) and *I*^*2*^ (*I*^*2*^ = 0.0%; 95% CI: [0.0%−51.1%]) statistics revealed no heterogeneity beyond chance between studies. Egger’s test revealed no publication bias (*p* = .41). A forest plot for pooled effects (pre-post differences across augmentation-control groups) for each study appears in Supplementary Information S2.2.

#### Subgroup Analyses

Given the small number of studies in our meta-analysis we conducted only two subgroup analyses. The first examined whether weight status was a potential moderator. The meta-analysis was repeated with studies coded as underweight (*n* = 6) and non-underweight (*n* = 11). Studies where a larger proportion of participants had a diagnosis of anorexia nervosa were coded as underweight. Results showed a significant effect of acute augmentations for both underweight (Hedge’s *g* = 0.15; 95% CI: [0.07, 0.23], *p* < .01) and non-underweight samples (Hedge’s *g* = 0.13; 95% CI: [−0.09, 0.36], *p* < .01), with small effect sizes. However, the non-underweight sample had 95% confidence intervals for the effect size that crossed zero. Cochrane’s *Q* and *I*^*2*^ statistics revealed no heterogeneity beyond chance for underweight samples (*Q* = 0.70; *I*^2^ = 0.0%) but mild heterogeneity for non-underweight samples (*Q* = 14.04; *I*^2^ = 28.8%). The second investigated TAU (*n* = 11) versus single therapy approaches (*n* = 6). There was a significant effect of acute augmentations for both TAU (Hedge’s *g* = 0.15; 95% CI: [0.02, 0.27], *p* < .01) and single therapy approaches (Hedge’s *g* = 0.12; 95% CI: [−0.27, 0.52], *p* < .01), with small effect sizes. Cochrane’s *Q* and *I*^*2*^ statistics revealed no heterogeneity beyond chance for TAU (*Q* = 6.60; *I*^2^ = 0.0%) but mild heterogeneity for single therapy approaches (*Q* = 8.13; *I*^2^ = 38.5%).

## Conclusions

This research was triggered by a review and meta-analysis of acute augmentations in psychological therapy across different mental health difficulties [10], which included only one study of people with eating disorders. Our systematic search identified 29 studies that met criteria for inclusion, with the earliest study published in 1989, examining the addition of response prevention to CBT [26]. Around one-third (*n* = 9) of these studies were published since 2020, indicating an increasing interest in acute augmentation in eating disorders driven by two factors. The first is the lack of recent progress in improving remission rates from current frontline outpatient treatments for eating disorders. The second is the evidence showing that early progress in treatment, across different treatments, treatment settings, diagnoses, and age groups, is the most robust predictor of outcome for people with eating disorders [11, 56, 57]. In the face of this slow progress there is emerging evidence to suggest that moving slower responders to a greater intensity of therapy can improve their outcomes commensurate to more rapid responders [58]. Acute augmentation represents a short-term “boost” to psychological therapy that may also allow slower responders an opportunity obtain the same outcomes as more rapid responders, thus improving outcomes in a more cost-effective way than moving to another treatment or using combination therapy.

While we identified subgroups of acute augmentations, none represented a large enough group to allow conclusions about the usefulness of specific augmentation approaches. We found acute augmentations were more effective than no augmentation in 72.4% of studies. These results provide preliminary support for use of acute augmentations to improve psychological therapy for eating disorders, but more studies are required to identify specific augmentations for different groups, which augmentations are best paired with which psychological therapies, and when augmentation is not beneficial.

In the meta-analysis, we obtained a small but significant between-group effect size favouring the addition of augmentation to psychological therapies for eating disorders, and this effect was present for both underweight and non-underweight populations, and for augmentation to single therapy approaches (e.g., CBT) versus TAU. Overall the results are robust in the face of no or low heterogeneity and no evidence of publication bias. The effect size (0.14) was a little smaller than that obtained in the meta-analysis across psychological disorders [10], 0.27, but that could be explained by the greater preponderance of biological augmentations in the latter which tended to produce the greater effect sizes. For example, no eating disorder studies using pharmacology were included; 20 were captured in the initial search but did not meet the definition of acute augmentation as they were combination therapy (i.e., medication prescribed alongside psychological therapy). This does suggest one avenue for future research.

There are three main limitations of the current research. Given the small number of RCTs included in the meta-analysis we did not include a risk-of-bias assessment. Second, it is not possible to make any conclusions about the type of augmentation that may be preferred, given that most acute augmentations included were only evaluated on one occasion (24.1% in the systematic review and 47.1% of RCTs). The papers included in the systematic review suggest that types of acute augmentation selected move through phases of popularity, with an initial focus on response prevention in the binge-eating disorders, to motivational interviewing in the mid-period, with more recent interest in the use of mobile phone Apps to boost impact and engagement. Augmentation studies of cognitive bias modification were sparse (*n* = 1), which suggests a future area of research given these were second most effective specific augmentation approaches after psychotropic drugs for psychological disorders [10]. Since conducting our search, one further study investigating cognitive bias modification as an acute augmentation has been published [59]. Third, we were unable to conduct an indepth subgroup analysis of the timing of acute augmentation due to too few studies in each subgroup.

Our observations from conducting this review and meta-analysis is that the impact of the vibrant field of eating disorders intervention research is being limited by an over-reliance on the traditional randomized controlled trial. Use of more innovative clinical trial designs that can enhance flexibility and improve efficiency of both resource allocation and participant involvement are required [60], especially for anorexia nervosa where we have failed to establish one therapy is better than another [9]. Acute augmentation designs offer a rigorous design for clinical trials to more quickly test interventions that could significantly enhance response and advance knowledge in the field. Our results suggest that this is a pathway worth pursuing.

### Supplementary Information

Below is the link to the electronic supplementary material.

Supplementary file1 (DOCX 167 KB)

## Data Availability

The data that support the findings of this study are available from the corresponding author upon reasonable request.
