# Supplementary material for: Acute Augmentations to Psychological Therapies in Eating Disorders: A Systematic Review and Meta-Analysis
Source: Curr Psychiatry Rep. 2024 Aug 2;26(9):447–59. doi: 10.1007/s11920-024-01519-y (PMC11344718; doi:10.1007/s11920-024-01519-y)
Supplement: Supplementary file 1 — Supplementary file1 (DOCX 167 KB) [file 11920_2024_1519_MOESM1_ESM.docx]

**Supplemental Online Content**

**Acute Augmentations to Psychological Therapies in Eating Disorders: A Systematic Review and Meta-Analysis**

Jamie-Lee Pennesi, PhD* ORCID: 0000-0003-1461-7303

Catherine Johnson, PhD ORCID: 0000-0003-2733-3326

Marcela Radunz, PhD ORCID: 0000-0002-2858-0313

Tracey D Wade, PhD ORCID: 0000-0003-4402-770X

Flinders University Institute for Mental Health and Wellbeing and Blackbird Initiative, Flinders University, South Australia, Australia **Supplementary Methods**

- 1. **Definitions**

Table S1. Detailed definitions

- 1. **Differences between acute augmentation therapy and other therapy approaches**

Table S2. Descriptions of different therapy approaches

- 1. **Deviations from protocol**

Table S3. Summary of deviations from PROSPERO protocol

- 1. **Search strategy**

Table S4. Summary of search terms

- 1. **Inclusion and exclusion criteria**
  2. **Excluded studies with reasons**

Table S5. Summary of excluded studies with brief reasons (*n* = 136)

1. **Supplementary Results**
   1. **Forest Plot**

Figure S1. Forest Plot of Pooled Effects (Hedges’ *g)* for each study

1. **Supplementary Methods**
   1. **Definitions**

**Table S1.** Detailed definitions.

| **Term** | **Definition** |
| --- | --- |
| Psychological therapy | Psychological therapy must be manualised. Psychological therapy can be delivered in a face-to-face (in-person) or online (virtual) format but must be at least partially clinician- or researcher-led (must not be entirely self-guided). The psychological therapy can be treatment-as-usual in the case that it is a manualised treatment. The psychological therapy can be inpatient or outpatient treatment. |
| Manualised therapy | Manualised therapies are those for which a specific treatment manual, guide, or protocol is utilised (e.g., cognitive behavioural therapy, dialectical behaviour therapy). In some cases, the treatment manual can be published and is cited by the study authors. The treatment can be delivered as part of a standard inpatient or day program, where all patients receive the same kind of treatment. The treatment can be individual or group-based treatment. Manualised therapy can be treatment-as-usual if all the participants are receiving some kind of treatment-as-usual. |
| Acute augmentations | An acute augmentation is an intervention that is delivered immediately before, during, or after a session of psychological therapy, with the aim of enhancing the therapeutic impact of the psychological therapy on treatment outcomes (e.g., reducing eating disorder symptoms) [1]. Acute augmentations can be delivered as a one off, but they can also be repeated across multiple therapy sessions over the course of therapy.  This is distinct from a combination treatment, in which two independent therapies or therapeutic approaches are delivered on a long-term basis in parallel to potentially provide additive benefits (e.g., medication prescribed alongside a course of psychological therapy) [1]. In contrast, acute augmentations target specific biological or psychological processes on at least one occasion to boost outcomes for an evidenced-based treatment (e.g., bias modification or imagery might be used to reduce social anxiety such that the therapeutic tasks can be better utilized by the patient [2,3]. An acute augmentation can be use of a smartphone application or a pharmacological intervention if it is delivered as an adjunct (e.g., before, during, or after exposure therapy sessions). |
| Treatment-as-usual | Treatment-as-usual is the same as usual care and “standard of care” in the real world and can include any variation of care or treatment (e.g., receiving eating disorder or non-eating disorder care, an inpatient treatment program, group-based outpatient treatment, a range of interventions, seeing a general practitioner). Treatment-as-usual can be a manualised treatment or control/comparator as long as all participants are receiving some kind of treatment-as-usual. |
| Comparator/control | Comparators can include treatment-as-usual, no treatment, waitlist control, sham condition, a placebo etc. Comparators including another psychological or active intervention (e.g., another psychological therapy, art therapy) will not be included. If a study contained more than one potential comparator/control, we selected the group that represented the control group/true comparator (i.e., psychological therapy but no augmentation or active intervention). Studies that did not include a control group/true comparator were excluded. |

- 1. **Differences between acute augmentation therapy and other therapy approaches**

**Table S2.** Descriptions of different therapy approaches.

| **Therapy approach** | **Brief description** |
| --- | --- |
| Acute augmentation | An intervention delivered immediately before, during, or after a session of psychological therapy, with the aim of enhancing the therapeutic impact of a single session of therapy [1], it can be delivered as a one off, but it can be repeated across multiple therapy sessions. |
| Stepped care | Treatment is delivered in a pre-determined sequenced manner based on the severity of patients’ symptoms and treatment response to earlier treatments [4] (e.g., an augmentation, a combination treatment, or a new treatment delivered to treatment non-responders following a course of psychological therapy). |
| Adaptive treatment | Treatment is adapted based on lack of treatment efficacy, disease progression, or safety [5] (e.g., augmentation delivered only to treatment non-responders). For summary of different adaptive design methodologies, see Edney and Pellizzer [6]. |
| Combination treatment | An intervention in which two independent therapies or therapeutic approaches are delivered on a long-term basis in parallel to potentially provide additive benefits (e.g., medication prescribed alongside a course of psychological therapy). |

- 1. **Deviations from protocol**

**Table S3.** Summary of deviations from PROSPERO protocol.

| **Protocol deviation** | **Brief description** |
| --- | --- |
| Inclusion criteria | The following criterion was added to the inclusion criteria: (1) the study was written in English. This had been mentioned in the search strategy but was not mentioned explicitly in the inclusion criteria. |
| Exclusion criteria | The following criteria were added to the exclusion criteria: (1) the study was a book or qualitative study; (2) the study was not focused on eating disorders, unless an eating disorder was co-occurring (e.g., obesity with an eating disorder); and (3) the study was still being conducted. These had not been mentioned explicitly in the exclusion criteria. |
| Definition of acute augmentation | In our original definition, we stated that an acute augmentation was a “…nonpharmacological outpatient intervention”; however, to more closely match the definition of acute augmentation used in Nord et al. [1], this phrase was removed. As a result, pharmacological and non-outpatient interventions were considered for inclusion. In addition, further details were added to our original definition of acute augmentations for clarity and to clearly distinguish acute augmentations from combination treatments. |
| Definition of psychological therapy | In our original definition, we did not explicitly mention that psychological therapy could also be treatment-as-usual. This was later added for clarity. |
| Definition of manualised therapy | A definition of manualised therapy was added for clarity. This had been briefly mentioned in parentheses but no formal definition had been provided. |
| Definition of treatment-as-usual | A definition of treatment-as-usual was added for clarity. This had been briefly mentioned but no formal definition had been provided. |
| Definition of comparator/control | Additional information was added to the definition of comparator/control for clarity (e.g., mention of comparators including another psychological or active intervention, mention of selection of control group in studies with more than one potential comparator/control). |
| Definition of combination treatment | Further details were added to our original definition of combination treatment for clarity and to clearly distinguish combination treatment from acute augmentations. |
| Primary outcome | In our original protocol, we stated that we would record the primary psychological outcome reported in each study or the first continuous outcome reported in the Results. A later decision was made to include only studies with eating disorder outcomes in the meta-analysis to ensure homogeneity across outcomes and maintain the focus on eating disorders. Studies that did not include eating disorder outcomes were included in the systematic review, but not in the meta-analysis. For studies that did not include an eating disorder outcome, the first primary psychological outcome reported in each study was used for calculation of within-group gain scores (for intervention efficacy). If means and standard deviations were not provided for a psychological outcome, another measure was used (e.g., frequency of binge episodes). |
| Meta-analysis | In our original protocol, we did not explicitly state that we would include only randomised trials in the meta-analysis. A later decision was made to do so to examine the most robust of augmentation studies. |
| Secondary analyses | In our original protocol, we stated that we would measure within-group effects by calculating the uncontrolled effect size (Hedges’ g) for the primary outcome at baseline and post-treatment and the 95% confidence intervals for all studies, as secondary analysis. These were not calculated or reported. |
| Risk-of-bias (quality) assessment | In our original protocol, we stated that we would assess risk-of-bias (quality) of included studies. This was not assessed given the small number of studies included in the meta-analysis. |

- 1. **Search strategy**

The present study was conducted and reported in line with evidence-based guidelines for reporting systematic reviews and meta-analyses [7,8]. The databases Medline (via Ovid), Scopus (via Elsevier), and PsycINFO (via Ovid) were searched for eligible studies for all years covered through to 29^th^ of January 2024, using the search terms presented in **Table S4** below. The search terms are based on those used by Nord et al. [1] but included a wider range of eating disorders and behaviours and terms related to psychological therapies and designs commonly used in eating disorders. Terms not related to eating disorders were removed. Search results were limited to English language papers. There were no restrictions placed on the date of publication. In addition, the ClinicalTrials.gov website was searched for unpublished studies, using the modified search terms in **Table S4** below.

**Table S4.** Summary of search terms.

| **Boolean operator** | **Search terms and term finders** |
| --- | --- |
| ***Medline via Ovid*** | |
|  | ("cognitive processing" OR "narrative exposure therapy" OR "prolonged exposure therapy" OR "virtual reality exposure therapy" OR "exposure therapy" OR "cognitive behav* therapy" OR "cognitive-behav* therapy" OR CBT OR "cognitive therapy" OR "eye movement desensiti*" OR EMDR OR "psychodynamic psychotherapy" OR psychodynamic OR "interpersonal psychotherapy" OR "mindfulness" OR "acceptance and commitment therapy" OR ACT OR “family based treat*” OR “family-based treat*” OR “family based therapy” OR “family-based therapy*” OR FBT OR "attention training" OR "dialectical behav* therapy" OR DBT OR “emotion* focused therapy” OR “emotion-focused therapy” OR EFT OR "animal assisted therapy" OR "animal-assisted therapy" OR "cognitive analytic* therapy" OR "imagery psychotherapy" OR "behav* activation" OR “narrative therapy” OR psychotherapy).ti,ab,kw. OR Cognitive Behavioral Therapy/ OR Narrative Therapy/ OR Eye Movement Desensitization Reprocessing/ OR Psychotherapy/ OR Imagery, Psychotherapy/ OR Interpersonal Psychotherapy/ OR Psychotherapy, Psychodynamic/ OR Mindfulness/ OR "Acceptance and Commitment Therapy"/ OR Animal Assisted Therapy/ OR Behavior Therapy/ OR Family Therapy/ OR Dialectical Behavior Therapy/ OR Emotion-Focused Therapy/ |
| AND | ("eating disorder*" OR anorexi* OR bulimi* OR "binge eat*" OR "binge-eat*" OR “other specified feeding or eating disorder” OR OSFED OR “eating disorder not otherwise specified” OR EDNOS OR “avoidant / restrictive food intake disorder” OR “avoidant restrictive food intake disorder” OR ARFID OR “disordered eat*” OR bing* OR restrict* OR fasting OR diet* OR “body image” OR “body dissatis*”).ti,ab,kw. OR Body Image/ OR Body Dissatisfaction/ OR "Feeding and Eating Disorders"/ |
| AND | (augment* OR enhanc* OR adjunct* OR conjunct* OR amplify OR assist* OR supplement* OR combin* OR adapt* OR “stepped care” OR “stepped-care”).ti,ab,kw. |
| AND | ("clinical trial*" OR "control* trial*" OR randomi* OR RCT OR "controlled before-after studies" OR "pilot project*" OR "feasibility trial*" OR "exploratory trial*" OR “feasibility” OR “acceptability” OR “effectiveness”).ti,ab. OR Clinical Trial/ OR Controlled Before-After Studies/ OR Randomized Controlled Trial/ OR Pilot Projects/ |
| ***Scopus via Elsevier*** | |
|  | TITLE-ABS-KEY("cognitive processing" OR "narrative exposure therapy" OR "prolonged exposure therapy" OR "virtual reality exposure therapy" OR "exposure therapy" OR "cognitive behav* therapy" OR "cognitive-behav* therapy" OR CBT OR "cognitive therapy" OR "eye movement desensiti*" OR EMDR OR "psychodynamic psychotherapy" OR psychodynamic OR "interpersonal psychotherapy" OR "mindfulness" OR "acceptance and commitment therapy" OR ACT OR “family based treat*” OR “family-based treat*” OR “family based therapy” OR “family-based therapy*” OR FBT OR "attention training" OR "dialectical behav* therapy" OR DBT OR “emotion* focused therapy” OR “emotion-focused therapy” OR EFT OR "animal assisted therapy" OR "animal-assisted therapy" OR "cognitive analytic* therapy" OR "imagery psychotherapy" OR "behav* activation" OR “narrative therapy” OR psychotherapy) |
| AND | TITLE-ABS-KEY ("eating disorder*" OR anorexi* OR bulimi* OR "binge eat*" OR "binge-eat*" OR “other specified feeding or eating disorder” OR OSFED OR “eating disorder not otherwise specified” OR EDNOS OR “avoidant / restrictive food intake disorder” OR “avoidant restrictive food intake disorder” OR ARFID OR “disordered eat*” OR bing* OR restrict* OR fasting OR diet* OR “body image” OR “body dissatis*”) |
| AND | TITLE-ABS-KEY (augment* OR enhanc* OR adjunct* OR conjunct* OR amplify OR assist* OR supplement* OR combin* OR adapt* OR “stepped care” OR “stepped-care”) |
| AND | TITLE-ABS ("clinical trial*" OR "control* trial*" OR randomi* OR RCT OR "controlled before-after studies" OR "pilot project*" OR "feasibility trial*" OR "exploratory trial*" OR “feasibility” OR “acceptability” OR “effectiveness”) |
| ***PsycINFO (via Ovid)*** | |
|  | ("cognitive processing" OR "narrative exposure therapy" OR "prolonged exposure therapy" OR "virtual reality exposure therapy" OR "exposure therapy" OR "cognitive behav* therapy" OR "cognitive-behav* therapy" OR CBT OR "cognitive therapy" OR "eye movement desensiti*" OR EMDR OR "psychodynamic psychotherapy" OR psychodynamic OR "interpersonal psychotherapy" OR "mindfulness" OR "acceptance and commitment therapy" OR ACT OR “family based treat*” OR “family-based treat*” OR “family based therapy” OR “family-based therapy*” OR FBT OR "attention training" OR "dialectical behav* therapy" OR DBT OR “emotion* focused therapy” OR “emotion-focused therapy” OR EFT OR "animal assisted therapy" OR "animal-assisted therapy" OR "cognitive analytic* therapy" OR "imagery psychotherapy" OR "behav* activation" OR “narrative therapy” OR psychotherapy).ti,ab. OR Cognitive Behavioral Therapy/ OR Narrative Therapy/ OR Eye Movement Desensitization Reprocessing/ OR Psychotherapy/ OR Imagery, Psychotherapy/ OR Interpersonal Psychotherapy/ OR Psychotherapy, Psychodynamic/ OR Mindfulness/ OR "Acceptance and Commitment Therapy"/ OR Animal Assisted Therapy/ OR Behavior Therapy/ OR Family Therapy/ OR Dialectical Behavior Therapy/ OR Emotion-Focused Therapy/ |
| AND | ("eating disorder*" OR anorexi* OR bulimi* OR "binge eat*" OR "binge-eat*" OR “other specified feeding or eating disorder” OR OSFED OR “eating disorder not otherwise specified” OR EDNOS OR “avoidant / restrictive food intake disorder” OR “avoidant restrictive food intake disorder” OR ARFID OR “disordered eat*” OR bing* OR restrict* OR fasting OR diet* OR “body image” OR “body dissatis*”).ti,ab. OR Body Image/ OR Body Dissatisfaction/ OR Eating Disorders/ |
| AND | (augment* OR enhanc* OR adjunct* OR conjunct* OR amplify OR assist* OR supplement* OR combin* OR adapt* OR “stepped care” OR “stepped-care”).ti,ab. |
| AND | ("clinical trial*" OR "control* trial*" OR randomi* OR RCT OR "controlled before-after studies" OR "pilot project*" OR "feasibility trial*" OR "exploratory trial*" OR “feasibility” OR “acceptability” OR “effectiveness”).ti,ab. OR Clinical Trials/ OR Randomized Controlled Trials/ |
| ***ClinicalTrials.gov*** | |
|  | [Condition or disease:] (eating disorder OR anorexia nervosa OR bulimia nervosa OR binge eating disorder OR binge-eating disorder OR binge eating OR binge-eating OR disordered eating) |
| AND | [Other terms:] (augment OR augmentation OR enhance OR adjunct OR conjunctive OR amplify OR assist OR supplement OR combine OR adapt OR adaptation OR stepped care OR stepped-care) |
| AND | [Intervention/treatment:] (cognitive processing OR narrative exposure therapy OR prolonged exposure therapy OR virtual reality exposure therapy OR exposure therapy OR cognitive behavioural therapy OR cognitive-behavioural therapy OR cognitive behavioral therapy OR cognitive-behavioral therapy OR cognitive behaviour therapy OR cognitive-behaviour therapy OR cognitive behavior therapy OR cognitive-behavior therapy OR cognitive behavioral therapy CBT OR CBT OR CBT-E OR cognitive therapy OR eye movement desensitization and reprocessing OR EMDR OR psychodynamic psychotherapy OR psychodynamic OR interpersonal psychotherapy OR mindfulness OR acceptance and commitment therapy OR ACT OR acceptance and commitment therapy, ACT OR family based treatment OR family-based treatment OR family-based treatment \(FBT\) OR family based therapy OR family-based therapy OR family-based therapy \(FBT\) OR FBT OR attention training OR dialectical behavioural therapy OR dialectical behavioral therapy OR dialectical behaviour therapy OR dialectical behavior therapy OR dialectical behavior therapy \(DBT\) OR DBT OR emotion focused therapy OR emotion-focused therapy OR emotionally focused therapy OR EFT OR animal assisted therapy OR animal-assisted therapy OR cognitive analytical therapy OR cognitive analytic therapy OR cognitive analytic therapy \(CAT\) OR imagery psychotherapy OR imagery OR behavioural activation OR behavioral activation OR narrative therapy OR psychotherapy) |

- 1. **Inclusion and exclusion criteria**

Eating disorders could include anorexia nervosa, bulimia nervosa, binge eating disorder, other specified feeding or eating disorder (DSM-V), eating disorder not otherwise specified (DSM-IV), or avoidant/restrictive food intake disorder. Eating disorder diagnosis did not have to be confirmed during enrolment or assessed as part of the study protocol if inclusion criteria for the study included criteria or diagnosis of an eating disorder and participants were recruited from an inpatient, outpatient, or specialist eating disorder centre. We included both clinical and subclinical eating disorders, given there is no difference in clinical impairment [9]. We did not include pica or rumination disorder diagnoses. Participants with comorbid diagnoses (e.g., major depressive disorder, social anxiety disorder) were not excluded. Unpublished studies were included if they had sufficient data to calculate effect sizes; we also conducted a search of Google Scholar for a published version of the study. Secondary or follow-up analysis papers (e.g., 1-year follow-up) were included; however, if the paper did not report on pre-treatment and end-of-treatment outcome data we conducted a search of Google Scholar for the original paper.

Also see definitions above for more information regarding psychological therapy, manualised therapy, acute augmentation, and comparator/control.

- 1. **Excluded studies with reasons**

**Table S5.** Summary of excluded studies with brief reasons (*n* = 136).

| **Authors (year of publication)** | **Reason for exclusion** |
| --- | --- |
| Accurso et al. (2018) [10] | No control/comparison group |
| Assistance Publique Hopitaux De Marseille (2018) [11]^a^ | Insufficient data |
| Balzan et al. (2023) [12] | No psychological therapy |
| Bauer et al. (2006) [13] | Combination treatment |
| Bauer et al. (2012) [14] | No psychological therapy |
| Bishop-Gilyard et al. (2011) [15] | No acute augmentation |
| Blomquist et al. (2011) [16] | No acute augmentation |
| Boston University (2013) [17]^a^ | No control/comparison group |
| Boutelle et al. (2023) [18] | No acute augmentation |
| Brockmeyer et al. (2014) [19] | No control/comparison group |
| Brockmeyer et al. (2021) [20] | No control/comparison group |
| Bulik et al. (1998) [21] | No control/comparison group |
| Bulik et al. (1998) [22] | Secondary data analysis |
| Cassin et al. (2008) [23] | Insufficient data |
| Chao et al. (2019) [24] | No eating disorder diagnosis |
| Children's Hospital Medical Center, Cincinnati (2020) [25]^a^ | Insufficient data |
| Children's Hospital of Philadelphia (2016) [26]^a^ | Insufficient data |
| Children's Hospital of Philadelphia (2021) [27]^a^ | Study ongoing |
| Children's Hospitals and Clinics of Minnesota (2015) [28]^a^ | No acute augmentation |
| Danielsen et al. (2016) [29] | No acute augmentation |
| Depestele et al. (2017) [30] | No control/comparison group |
| Devlin et al. (2000) [31] | No acute augmentation |
| Devlin et al. (2005) [32] | Combination treatment |
| Devlin et al. (2007) [33] | Secondary data analysis |
| Drexel University (2006) [34]^a^ | Insufficient data |
| Drexel University (2019) [35]^a^ | Insufficient data |
| Drexel University (2020) [36]^a^ | Insufficient data |
| Drexel University (2022) [37]^a^ | Study ongoing |
| Duke University (2016) [38]^a^ | No eating disorder diagnosis |
| Duke University (2023) [39]^a^ | Study ongoing |
| Eichen et al. (2023) [40] | Integrates two treatments |
| Eldredge et al. (1997) [41] | No acute augmentation |
| Ellison et al. (2016) [42] | No acute augmentation |
| Estey et al. (2022) [43] | No acute augmentation |
| Fichter et al. (2012) [44] | Not focused on treatment |
| Gendron et al. (1992) [45] | No psychological therapy |
| Giombini et al. (2022) [46] | Insufficient data |
| Girz et al. (2013) [47] | No acute augmentation |
| Godart et al. (2012) [48] | Combination treatment |
| Goldberg et al. (1979) [49] | Combination treatment |
| Goldboom et al. (1997) [50] | Combination treatment |
| Gordon et al. (2021) [51] | No continuous psychological outcome |
| Gorin et al. (2003) [52] | No acute augmentation |
| Gowers et al. (2010) [53] | No acute augmentation |
| Grammer et al. (2022) [54] | Integrates two treatments |
| Grilo & Masheb (2007) [55] | Combination treatment |
| Grilo et al. (2012) [56] | Combination treatment |
| Grilo et al. (2014) [57] | Combination treatment |
| Grilo et al. (2022) [58] | Combination treatment |
| Hay et al. (2022) [59] | No acute augmentation |
| Herzog et al. (2022) [60] | No acute augmentation |
| Hildebrandt et al. (2012) [61] | No control/comparison group |
| Hildebrandt et al. (2017) [62] | Insufficient data |
| Hildebrandt et al. (2020) [63] | Insufficient data |
| Hospital de Clinicas de Porto Alegre (2019) [64]^a^ | Insufficient data |
| Hsu et al. (2001) [65] | No acute augmentation |
| Istituto Auxologico Italiano (2023) [66]^a^ | Study ongoing |
| Jacobi et al. (2002) [67] | Combination treatment |
| Jacobi et al. (2017) [68] | No continuous psychological outcome |
| Juarascio et al. (2021) [69] | Review, meta-analysis, case report, case series, commentary, editorial, study protocol, conference abstract, animal study, book, or qualitative study |
| Juarascio et al. (2023) [70] | Review, meta-analysis, case report, case series, commentary, editorial, study protocol, conference abstract, animal study, book, or qualitative study |
| Kelly et al. (2017) [71] | Insufficient data |
| Kim et al. (2019) [72] | No control/comparison group |
| King's College London (2018) [73]^a^ | Insufficient data |
| Kolar et al. (2017) [74] | Review, meta-analysis, case report, case series, commentary, editorial, study protocol, conference abstract, animal study, book, or qualitative study |
| Korrelboom et al. (2009) [75] | Stepped care or adaptive design |
| Lanzarone et al. (2014) [76] | Combination treatment |
| Leitenberg et al. (1994) [77] | Combination treatment |
| Levinson et al. (2015) [78] | Insufficient data |
| Lock et al. (2018) [79] | No control/comparison group |
| Lock et al. (2023) [80] | Stepped care or adaptive design |
| Lykos Therapeutics (2024) [81]^a^ | Study ongoing |
| MacDonald et al. (2017) [82] | No control/comparison group |
| MacDonald et al. (2021) [83] | Secondary data analysis |
| Manasse et al. (2020) [84] | Review, meta-analysis, case report, case series, commentary, editorial, study protocol, conference abstract, animal study, book, or qualitative study |
| Masheb et al. (2011) [85] | No control/comparison group |
| McCabe et al. (2008) [86] | Not focused on eating disorders |
| McIntosh et al. (2016) [87] | Integrates two treatments |
| Mental Health Services in the Capital Region, Denmark (2019) [88]^a^ | Insufficient data |
| Mills et al. (2023) [89] | No control/comparison group |
| Mitchell et al. (1990) [90] | Combination treatment |
| Molinari et al. (2005) [91] | Combination treatment |
| Naab et al. (2013) [92] | No acute augmentation |
| Nagoya City University (2023) [93]^a^ | Study ongoing |
| Neumayr et al. (2019) [94] | Insufficient data |
| Newbridge House (2016) [95]^a^ | Insufficient data |
| Newbridge House (2018) [96]^a^ | Insufficient data |
| Newbridge House (2020) [97]^a^ | Insufficient data |
| Nova Scotia Health Authority (2014) [98]^a^ | Insufficient data |
| Nyman-Carlsson et al. (2020) [99] | No acute augmentation |
| Palmer et al. (2002) [100] | No acute augmentation |
| Pauli et al. (2022) [101] | Insufficient data |
| Porra-Garcia et al. (2021) [102] | Insufficient data |
| Psychiatric University Hospital, Zurich (2022) [103]^a^ | Study ongoing |
| Recovery Record Research (2016) [104]^a^ | No psychological therapy |
| Reyes-Rodriguez (2010) [105]^a^ | Insufficient data |
| Rhodes et al. (2008) [106] | Insufficient data |
| Riva et al. (2003) [107] | No acute augmentation |
| Ruggeri (2017) [108]^a^ | Insufficient data |
| Shalvata Mental Health Center (2015) [109]^a^ | Insufficient data |
| Shalvata Mental Health Center (2023) [110]^a^ | Study ongoing |
| Shanghai Mental Health Center (2018) [111]^a^ | Insufficient data |
| Sheba Medical Center (2014) [112]^a^ | Insufficient data |
| Shingleton et al. (2016) [113] | No control/comparison group |
| St. Joseph's Healthcare Hamilton (2016) [114]^a^ | Study ongoing |
| Stanford University (2007) [115]^a^ | Insufficient data |
| Stanford University (2008) [116]^a^ | Insufficient data |
| Steinglass et al. (2014) [117] | No psychological therapy |
| Tanaka et al. (2006) [118] | Combination treatment |
| Thompson-Brenner et al. (2019) [119] | No control/comparison group |
| Timko et al. (2021) [120] | Review, meta-analysis, case report, case series, commentary, editorial, study protocol, conference abstract, animal study, book, or qualitative study |
| Treasure et al. (1999) [121] | No control/comparison group |
| Tronieri et al. (2020) [122] | Combination treatment |
| Universitaire Ziekenhuizen KU Leuven (2018) [123]^a^ | Insufficient data |
| University Health Network, Toronto (2015) [123]^a^ | Integrates two treatments |
| University of Barcelona (2015) [125]^a^ | Stepped care or adaptive design |
| University of Barcelona (2021) [126]^a^ | Study ongoing |
| University of Barcelona (2024) [127]^a^ | Study ongoing |
| University of California, San Diego (2020) [128]^a^ | Integrates two treatments |
| University of Edinburgh (2016) [129]^a^ | Insufficient data |
| University of North Carolina, Chapel Hill (2012) [130]^a^ | Insufficient data |
| University of North Carolina, Charlotte (2023) [131]^a^ | Study ongoing |
| University of Turin, Italy (2010) [132]^a^ | Combination treatment |
| VA Office of Research and Development (2018) [133]^a^ | Insufficient data |
| Van Passel et al. (2020) [134] | Insufficient data |
| Vander Wal et al. (2015) [135] | Insufficient data |
| Vandereycken et al. (1982) [136] | Combination treatment |
| Ventura et al. (1999) [137] | No continuous psychological outcome |
| Walsh et al. (1997) [138] | Combination treatment |
| Walsh et al. (2004) [139] | Combination treatment |
| Wang & Xiao (2021) [140] | Insufficient data |
| Washington University School of Medicine (2022) [141]^a^ | Study ongoing |
| Wilks (2006) [142] | Insufficient data |
| Wnuk (2011) [143] | No acute augmentation |
| Woolhouse et al. (2012) [144] | No acute augmentation |
| Yale University (2017) [145]^a^ | Stepped care or adaptive design |

^a^ author represents clinical trial sponsor as reported on ClinicalTrials.gov as does not report author(s).

1. **Supplementary Results**
   1. **Forest Plot**

**Figure S1.** Forest plot of pooled effects (Hedges’ *g)* for each study.


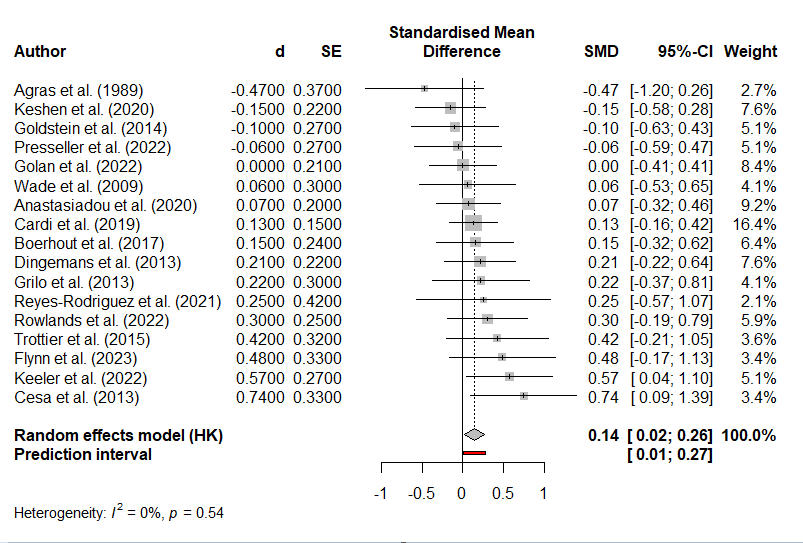


**References**

1. Nord CL, Longley B, Dercon Q, Phillips V, Funk J, Gormley S, et al. A transdiagnostic meta-analysis of acute augmentations to psychological therapy. *Nat Mental Health*. 2023;1: 389–401. https://doi.org/10.1038/s44220-023-00048-6.
2. Lazarov A, Marom S, Yahalom N, Pine DS, Hermesh H, Bar-Haim Y. Attention bias modification augments cognitive–behavioral group therapy for social anxiety disorder: A randomized controlled trial. *Psychol Med*. 2018;48(13): 2177–2185. https://doi.org/10.1017/S003329171700366X
3. McEvoy PM, Hyett MP, Bank SR, Erceg-Hurn D, Johnson AR, Kyron MJ, et al. Imagery-enhanced v. verbally-based group cognitive behavior therapy for social anxiety disorder: A randomized clinical trial. *Psychol Med*. 2022 05;52(7): 1277-1286. https://doi.org/10.1017/S0033291720003001
4. Rivero-Santana A, Perestelo-Perez L, Alvarez-Perez Y, Ramos-Garcia V, Duarte-Diaz A, Linertova R, et al. Stepped care for the treatment of depression: A systematic review and meta-analysis. *J Affect Disord*. 2021;294: 391–409. https://doi.org/10.1016/j.jad.2021.07.008
5. Chow SC, Chang M. Adaptive design methods in clinical trials: A review. *Orphanet J Rare Dis*. 2008;3(1): 1–13. https://doi.org/10.1186/1750-1172-3-11
6. Edney LC, Pellizzer ML. Adaptive design trials in eating disorder research: A scoping review. *Int J Eating Disord*. [Advance online publication] 2024, https://doi.org/10.1002/eat.24198
7. Moher D, Liberati A, Tetzlaff J, Altman DG, the PRISMA Group. Preferred reporting items for systematic reviews and meta-analyses: The PRISMA statement. *Ann Intern Med*. 2009;151(4): 264–269. https://doi.org/10.7326/0003-4819-151-4-200908180-00135
8. Page MJ, Moher D, Bossuyt PM, Boutron I, Hoffmann TC, Mulrow CD, et al. PRISMA 2020 explanation and elaboration: updated guidance and exemplars for reporting systematic reviews. *BMJ*. 2021;372: n160. https://doi.org/10.1136/bmj.n160
9. Wilkop M, Wade TD, Keegan E, Cohen-Woods S. Impairments among DSM-5 eating disorders: A systematic review and multilevel meta-analysis. *Clin Psychol Rev*. 2023;101: 102267. https://doi.org/10.1016/j.cpr.2023.102267
10. Accurso EC, Astrachan-Fletcher E, O'Brien S, McClanahan SF, Le Grange D. Adaptation and implementation of family-based treatment enhanced with dialectical behavior therapy skills for anorexia nervosa in community-based specialist clinics. *Eating Disord*. 2018;26(2): 149–63. https://dx.doi.org/10.1080/10640266.2017.1330319
11. Assistance Publique Hopitaux De Marseille. *Contribution of a virtual reality program in the treatment of dysmorphophobia for adolescent female with anorexia nervosa*. Identifier NCT03426930. U.S. National Library of Medicine; 2018. https://classic.clinicaltrials.gov/show/NCT03426930
12. Balzan RP, Gilder M, Thompson M, Wade TD. A randomized controlled feasibility trial of metacognitive training with adolescents receiving treatment for anorexia nervosa. *Int J Eating Disord*. 2023;56(9): 1820–1825. https://dx.doi.org/10.1002/eat.24009
13. Bauer C, Fischer A, Keller U. Effect of sibutramine and of cognitive-behavioural weight loss therapy in obesity and subclinical binge eating disorder. *Diabetes Obes Metab*. 2006;8(3): 289–295. https://dx.doi.org/10.1111/j.1463–1326.2005.00504.x
14. Bauer S, Okon E, Meermann R, Kordy H. Technology-enhanced maintenance of treatment gains in eating disorders: Efficacy of an intervention delivered via text messaging. *J Consult Clin Psychol*. 2012;80(4): 700–706. https://dx.doi.org/10.1037/a0028030
15. Bishop-Gilyard CT, Berkowitz RI, Wadden TA, Gehrman CA, Cronquist JL, Moore RH. Weight reduction in obese adolescents with and without binge eating. *Obes*. 2011;19(5): 982–987. https://dx.doi.org/10.1038/oby.2010.249
16. Blomquist KK, Grilo CM. Predictive significance of changes in dietary restraint in obese patients with binge eating disorder during treatment. *Int J Eating Disord*. 2011;44(6): 515–523. https://dx.doi.org/10.1002/eat.20849
17. Boston University. *A novel motivational ecological momentary intervention for anorexia nervosa*. Identifier NCT02076854. U.S. National Library of Medicine; 2013. https://classic.clinicaltrials.gov/show/NCT02076854.
18. Boutelle KN, Afari N, Obayashi S, Eichen DM, Strong DR, Peterson CB. Design of the CHARGE study: A randomized control trial evaluating a novel treatment for Veterans with binge eating disorder and overweight and obesity. *Contemp Clin Trials*. 2023;130(101242342): 107234. https://dx.doi.org/10.1016/j.cct.2023.107234
19. Brockmeyer T, Ingenerf K, Walther S, Wild B, Hartmann M, Herzog W, et al. Training cognitive flexibility in patients with anorexia nervosa: A pilot randomized controlled trial of cognitive remediation therapy. *Int J Eating Disord*. 2014;47(1): 24–31. https://dx.doi.org/10.1002/eat.22206
20. Brockmeyer T, Schmidt H, Leiteritz-Rausch A, Zimmermann J, Wunsch-Leiteritz W, Leiteritz A, Friederich H-C. Cognitive remediation therapy in anorexia nervosa: A randomized clinical trial. *J Consult Clin Psychol*. 2021;89(10): 805–815. https://dx.doi.org/10.1037/ccp0000675
21. Bulik CM, Sullivan PF, Carter FA, McIntosh VV, Joyce PR. The role of exposure with response prevention in the cognitive-behavioural therapy for bulimia nervosa. *Psychol Med*. 1998;28(3): 611–23. https://doi.org/10.1017/S0033291798006618
22. Bulik CM, Sullivan PF, Joyce PR, Carter FA, McIntosh VV. Predictors of 1-year treatment outcome in bulimia nervosa. *Compr Psychiatry*. 1998;39(4): 206–214. https://dx.doi.org/10.1016/S0010-440X(98)90062-1
23. Cassin SE, von Ranson KM, Heng K, Brar J, Wojtowicz AE. Adapted motivational interviewing for women with binge eating disorder: a randomized controlled trial. *Psychol Addict Behav*. 2008;22(3): 417–425. https://dx.doi.org/10.1037/0893-164X.22.3.417
24. Chao AM, Wadden TA, Walsh OA, Gruber KA, Alamuddin N, Berkowitz RI, Tronieri JS. Effects of liraglutide and behavioral weight loss on food cravings, eating behaviors, and eating disorder psychopathology. *Obes*. 2019;27(12): 2005–2010. https://dx.doi.org/10.1002/oby.22653
25. Children's Hospital Medical Center, Cincinnati. *Parent emotion coaching for anorexia nervosa*. Identifier NCT04421989. U.S. National Library of Medicine; 2020. https://classic.clinicaltrials.gov/show/NCT04421989.
26. Children's Hospital of Philadelphia. *Cognitive remediation therapy for adolescents with anorexia nervosa*. Identifier NCT02883413. U.S. National Library of Medicine; 2016. https://classic.clinicaltrials.gov/show/NCT02883413.
27. Children's Hospital of Philadelphia. Shifting perspectives R33 phase: *Enhancing outcomes in anorexia nervosa with CRT*. Identifier NCT05017831. U.S. National Library of Medicine; 2021. https://classic.clinicaltrials.gov/show/NCT05017831.
28. Children's Hospitals and Clinics of Minnesota. *Treatment outcome in eating disorders*. Identifier NCT03599921. U.S. National Library of Medicine; 2015. https://classic.clinicaltrials.gov/show/NCT03599921.
29. Danielsen YS, Ardal Rekkedal G, Frostad S, Kessler U. Effectiveness of enhanced cognitive behavioral therapy (CBT–E) in the treatment of anorexia nervosa: A prospective multidisciplinary study. *BMC Psychiatry*. 2016;16(1): 342. https://doi.org/10.1186/s12888-016-1056-6
30. Depestele L, Claes L, Dierckx E, Colman R, Schoevaerts K, Lemmens GMD. An adjunctive multi-family group intervention with or without patient participation during an inpatient treatment for adolescents with an eating disorder: A pilot study. *Eur Eating Disord Rev*. 2017;25(6): 570–578. https://dx.doi.org/10.1002/erv.2556
31. Devlin MJ, Goldfein JA, Carino JS, Wolk SL. Open treatment of overweight binge eaters with phentermine and fluoxetine as an adjunct to cognitive-behavioral therapy. *Int J Eating Disord*. 2000;28(3): 325–332. https://dx.doi.org/10.1002/1098-108X(200011)28:3<325::AID-EAT10>3.0.CO;2-3
32. Devlin MJ, Goldfein JA, Petkova E, Jiang H, Raizman PS, Wolk S, et al. Cognitive behavioral therapy and fluoxetine as adjuncts to group behavioral therapy for binge eating disorder. *Obes Res*. 2005;13(6): 1077–1088. https://doi.org/10.1038/oby.2005.126
33. Devlin MJ, Goldfein JA, Petkova E, Linxu L, Walsh BT. Cognitive behavioral therapy and fluoxetine for binge eating disorder: Two-year follow-up. *Obes*. 2007;15(7): 1702–1709. https://dx.doi.org/10.1038/oby.2007.203
34. Drexel University. *Effectiveness of a normalization of eating intervention program for treating women with eating disorders*. Identifier NCT00272545. U.S. National Library of Medicine; 2006. https://classic.clinicaltrials.gov/show/NCT00272545.
35. Drexel University. *Augmenting cognitive behavioral therapy with inhibitory control training*. Identifier NCT04076553. U.S. National Library of Medicine; 2019. https://classic.clinicaltrials.gov/show/NCT04076553.
36. Drexel University. *Optimizing mindfulness and acceptance based behavioral treatment for bulimia nervosa and binge eating disorder*. Identifier NCT04625959. U.S. National Library of Medicine; 2020. https://classic.clinicaltrials.gov/show/NCT04625959.
37. Drexel University. *Identifying effective technological-based augmentations to enhance outcomes from self-help cognitive behavior therapy for binge eating*. Identifier NCT05937243. U.S. National Library of Medicine; 2022. https://classic.clinicaltrials.gov/show/NCT05937243.
38. Duke University. *Neuromodulation enhanced cognitive restructuring: A proof of concept study*. Identifier NCT02573246. U.S. National Library of Medicine; 2016. https://classic.clinicaltrials.gov/show/NCT02573246.
39. Eichen DM, Strong DR, Twamley EW, Boutelle KN. Adding executive function training to cognitive behavioral therapy for binge eating disorder: A pilot randomized controlled trial. *Eat Behav*. 2023;51(101090048):101806. https://dx.doi.org/10.1016/j.eatbeh.2023.101806
40. Eldredge KL, Stewart Agras W, Arnow B, Telch CF, Bell S, Castonguay L, Marnell M. The effects of extending cognitive-behavioral therapy for binge eating disorder among initial treatment nonresponders. *Int J Eating Disord*
41. . 1997;21(4) :347–352. https://dx.doi.org/10.1002/(SICI)1098-108X(1997)21:4<347::AID-EAT7>3.0.CO;2-O
42. Ellison JM, Simonich HK, Wonderlich SA, Crosby RD, Cao L, Mitchell JE, et al. Meal patterning in the treatment of bulimia nervosa. *Eat Behav*. 2016;20: 39–42. https://dx.doi.org/10.1016/j.eatbeh.2015.11.008
43. Estey EEE, Roff C, Kozlowski MB, Rovig S, Guyker WM, Cook-Cottone CP. Efficacy of Eat Breathe Thrive: A randomized controlled trial of a yoga-based program. *Body Image*. 2022;42(101222431): 427–439. https://dx.doi.org/10.1016/j.bodyim.2022.07.009
44. Fichter MM, Quadflieg N, Nisslmuller K, Lindner S, Osen B, Huber T, Wunsch-Leiteritz W. Does internet-based prevention reduce the risk of relapse for anorexia nervosa? *Behav Res Ther*. 2012;50(3): 180–190. https://dx.doi.org/10.1016/j.brat.2011.12.003
45. Gendron M, Lemberg R, Allender J, Bohanske J. Effectiveness of the intensive group process-retreat model in the treatment of bulimia. *Group*. 1992;16(2): 69–78. https://dx.doi.org/10.1007/BF01459706
46. Giombini L, Nesbitt S, Kusosa R, Fabian C, Sharia T, Easter A, Tchanturia K. Neuropsychological and clinical findings of cognitive remediation therapy feasibility randomised controlled trial in young people with anorexia nervosa. *Eur Eating Disord Rev*. 2022;30(1): 50–60. https://dx.doi.org/10.1002/erv.2874
47. Girz L, Robinson AL, Foroughe M, Jasper K, Boachie A. Adapting family-based therapy to a day hospital programme for adolescents with eating disorders: Preliminary outcomes and trajectories of change. *J Fam Ther*. 2013;35(Suppl 1): 102–120. https://dx.doi.org/10.1111/j.1467-6427.2012.00618.x
48. Godart N, Berthoz S, Curt F, Perdereau F, Rein Z, Wallier J, et al. A randomized controlled trial of adjunctive family therapy and treatment as usual following inpatient treatment for anorexia nervosa adolescents. *PloS One*. 2012;7(1): e28249. https://dx.doi.org/10.1371/journal.pone.0028249
49. Goldberg SC, Halmi KA, Eckert ED, Casper RC, Davis JM. Cyproheptadine in anorexia nervosa. *Br J Psychiatry*. 1979;134(0342367, b1k): 67–70. https://dx.doi.org/10.1192/bjp.134.1.67
50. Goldbloom DS, Olmsted M, Davis R, Clewes J, Heinmaa M, Rockert W, Shaw B. A randomized controlled trial of fluoxetine and cognitive behavioral therapy for bulimia nervosa: Short-term outcome. *Behav Res Ther*. 1997;35(9): 803–811. https://dx.doi.org/10.1016/S0005-7967(97)00041-7
51. Gordon G, Williamson G, Gkofa V, Schmidt U, Brockmeyer T, Campbell I. Participants' experience of approach bias modification training with transcranial direct current stimulation as a combination treatment for binge eating disorder. *Eur Eating Disord Rev*. 2021;29(6): 969–984. https://dx.doi.org/10.1002/erv.2859
52. Gorin AA, Le Grange D, Stone AA. Effectiveness of spouse involvement in cognitive behavioral therapy for binge eating disorder. *Int J Eating Disord*. 2003;33(4): 421–433. https://dx.doi.org/10.1002/eat.10152
53. Gowers SG, Clark AF, Roberts C, Byford S, Barrett B, Griffiths A, et al. A randomised controlled multicentre trial of treatments for adolescent anorexia nervosa including assessment of cost-effectiveness and patient acceptability: The TOuCAN trial. *HTA*. 2010;14(15). https://dx.doi.org/10.3310/hta14150
54. Grammer AC, Monterubio GE, D'Adamo L, Balantekin KN, Taylor CB, Fitzsimmons-Craft EE, Wilfley DE. Evaluation of a combined, online intervention for binge-type eating disorders and high body weight in young adults. *Eat Behav*. 2023;50(101090048): 101789. https://dx.doi.org/10.1016/j.eatbeh.2023.101789
55. Grilo CM, Masheb RM. Rapid response predicts binge eating and weight loss in binge eating disorder: Findings from a controlled trial of orlistat with guided self-help cognitive behavioral therapy. *Behav Res Ther*. 2007;45(11): 2537–2550. https://dx.doi.org/10.1016/j.brat.2007.05.010
56. Grilo CM, Masheb RM, Crosby RD. Predictors and moderators of response to cognitive behavioral therapy and medication for the treatment of binge eating disorder. *J Consult Clin Psychol*. 2012;80(5): 897–906. https://dx.doi.org/10.1037/a0027001
57. Grilo CM, Masheb RM, White MA, Gueorguieva R, Barnes RD, Walsh BT, et al. Treatment of binge eating disorder in racially and ethnically diverse obese patients in primary care: Randomized placebo-controlled clinical trial of self-help and medication. *Behav Res Ther*. 2014;58(9kp, 0372477): 1–9. https://dx.doi.org/10.1016/j.brat.2014.04.002
58. Grilo CM, Lydecker JA, Fineberg SK, Moreno JO, Ivezaj V, Gueorguieva R. Naltrexone-bupropion and behavior therapy, alone and combined, for binge-eating disorder: Randomized double-blind placebo-controlled trial. *Am J Psychiatry*. 2022;179(12): 927–937. https://dx.doi.org/10.1176/appi.ajp.20220267
59. Hay P, Palavras MA, da Luz FQ, Dos Anjos Garnes S, Sainsbury A, Touyz S, et al. Physical and mental health outcomes of an integrated cognitive behavioural and weight management therapy for people with an eating disorder characterized by binge eating and a high body mass index: A randomized controlled trial. *BMC Psychiatry*. 2022;22(1): 355. https://dx.doi.org/10.1186/s12888-022-04005-y
60. Herzog W, Wild B, Giel KE, Junne F, Friederich H–C, Resmark G, et al. Focal psychodynamic therapy, cognitive behaviour therapy, and optimised treatment as usual in female outpatients with anorexia nervosa (ANTOP study): 5-Year follow-up of a randomised controlled trial in Germany. *Lancet Psychiatry*. 2022;9(4): 280–290. https://dx.doi.org/10.1016/S2215-0366(22)00028-1
61. Hildebrandt T, Loeb K, Troupe S, Delinsky S. Adjunctive mirror exposure for eating disorders: A randomized controlled pilot study. *Behav Res Ther*. 2012;50(12): 797–804. https://dx.doi.org/10.1016/j.brat.2012.09.004
62. Hildebrandt T, Michaelides A, Mackinnon D, Greif R, DeBar L, Sysko R. Randomized controlled trial comparing smartphone assisted versus traditional guided self-help for adults with binge eating. *Int J Eating Disord*. 2017;50(11): 1313–1322. https://dx.doi.org/10.1002/eat.22781
63. Hildebrandt T, Michaeledes A, Mayhew M, Greif R, Sysko R, Toro-Ramos T, DeBar L. Randomized controlled trial comparing health coach-delivered smartphone-guided self-help with standard care for adults with binge eating. *Am J Psychiatry*. 2020;177(2): 134–142. https://dx.doi.org/10.1176/appi.ajp.2019.19020184
64. Hospital de Clinicas de Porto Alegre. *Effect of nutritional counseling associated with transcranial direct-current stimulation in binge eating reduction*. Identifier NCT04226794. U.S. National Library of Medicine; 2019. https://classic.clinicaltrials.gov/show/NCT04226794.
65. Hsu LK, Rand W, Sullivan S, Liu DW, Mulliken B, McDonagh B, Kaye WH. Cognitive therapy, nutritional therapy and their combination in the treatment of bulimia nervosa. *Psychol Med*. 2001;31(5): 871–879. https://dx.doi.org/10.1017/S003329170100410X
66. Istituto Auxologico Italiano. *The CORTEX randomized control trial*. Identifier NCT05912036. U.S. National Library of Medicine; 2023. https://classic.clinicaltrials.gov/show/NCT05912036.
67. Jacobi C, Dahme B, Dittmann R. Cognitive-behavioural, fluoxetine and combined treatment for bulimia nervosa: Short- and long-term results. *Eur Eating Disord Rev.* 2002;10(3): 179–198. https://dx.doi.org/10.1002/erv.452
68. Jacobi C, Beintner I, Fittig E, Trockel M, Braks K, Schade-Brittinger C, Dempfle A. Web-based aftercare for women with bulimia nervosa following inpatient treatment: Randomized controlled efficacy trial. *J Med Internet Res*. 2017;19(9): e321. https://dx.doi.org/10.2196/jmir.7668
69. Juarascio AS, Felonis CR, Manasse SM, Srivastava P, Boyajian L, Forman EM, Zhang F. The project COMPASS protocol: Optimizing mindfulness and acceptance-based behavioral treatment for binge-eating spectrum disorders. *Int J Eating Disord*. 2021;54(3): 451–458. https://dx.doi.org/10.1002/eat.23426
70. Juarascio AS, Presseller EK, Trainor C, Boda S, Manasse SM, Srivastava P, et al. Optimizing digital health technologies to improve therapeutic skill use and acquisition alongside enhanced cognitive-behavior therapy for binge-spectrum eating disorders: Protocol for a randomized controlled trial. *Int J Eating Disord*. 2023;56(2): 470–477. https://dx.doi.org/10.1002/eat.23864
71. Kelly AC, Wisniewski L, Martin-Wagar C, Hoffman E. Group-based compassion-focused therapy as an adjunct to outpatient treatment for eating disorders: A pilot randomized controlled trial. *Clin Psychol Psychot*. 2017;24(2): 475–487. https://dx.doi.org/10.1002/cpp.2018
72. Kim Y-R, Cardi V, Lee GY, An S, Kim J, Kwon G, et al. Mobile self-help interventions as augmentation therapy for patients with anorexia nervosa. *Telemed J E Health*. 2019;25(8): 740–747. https://dx.doi.org/10.1089/tmj.2018.0180
73. King's College London. *Social information processing in adolescents with eating disorders*. Identifier NCT03563755. U.S. National Library of Medicine; 2018. https://classic.clinicaltrials.gov/show/NCT03563755.
74. Kolar DR, Hammerle F, Jenetzky E, Huss M. Smartphone-enhanced low-threshold intervention for adolescents with anorexia nervosa (SELTIAN) waiting for outpatient psychotherapy: Study protocol of a randomised controlled trial. *BMJ Open*. 2017;7(10): e018049. https://dx.doi.org/10.1136/bmjopen-2017-018049
75. Korrelboom K, de Jong M, Huijbrechts I, Daansen P. Competitive memory training (COMET) for treating low self-esteem in patients with eating disorders: A randomized clinical trial. *J Consult Clin Psychol*. 2009;77(5): 974–980. https://dx.doi.org/10.1037/a0016742
76. Lanzarone C, Cuzzocrea F, Larcan R, Bongiorno A, Minì V. Effectiveness of cognitive behavioural psychotherapy alone and combined with pharmacotherapy in binge eating disorder: A differential research. *Br J Med Pract*. 2014;7(3): a724.
77. Leitenberg H, Rosen JC, Wolf J, Vara LS, Detzer MJ, Srebnik D. Comparison of cognitive-behavior therapy and desipramine in the treatment of bulimia nervosa. *Behav Res Ther*. 1994;32(1): 37–45. https://dx.doi.org/10.1016/0005-7967(94)90082-5
78. Levinson CA, Rodebaugh TL, Fewell L, Kass AE, Riley EN, Stark L, et al. D-Cycloserine facilitation of exposure therapy improves weight regain in patients with anorexia nervosa: A pilot randomized controlled trial. *JCP*. 2015;76(6): e787–e793. https://dx.doi.org/10.4088/JCP.14m09299
79. Lock J, Fitzpatrick KK, Agras WS, Weinbach N, Jo B. Feasibility study combining art therapy or cognitive remediation therapy with family-based treatment for adolescent anorexia nervosa. *Eur Eating Disord Rev*. 2018;26(1): 62–68. https://dx.doi.org/10.1002/erv.2571
80. Lock JD, Le Grange D, Bohon C, Matheson B, Jo B. Who responds to an adaptive intervention for adolescents with anorexia nervosa being treated with family-based treatment? Outcomes from a randomized clinical trial. *J Am Acad Child Adolesc Psychiatry*. 2023(hg5, 8704565). https://dx.doi.org/10.1016/j.jaac.2023.10.012
81. Lykos Therapeutics. *A multi-site study of MDMA-assisted psychotherapy for eating disorders*. Identifier NCT04454684. U.S. National Library of Medicine; 2024. https://classic.clinicaltrials.gov/show/NCT04454684.
82. MacDonald DE, McFarlane TL, Dionne MM, David L, Olmsted MP. Rapid response to intensive treatment for bulimia nervosa and purging disorder: A randomized controlled trial of a CBT intervention to facilitate early behavior change. *J Consult Clin Psychol*. 2017;85(9): 896–908. https://dx.doi.org/10.1037/ccp0000221
83. MacDonald DE, McFarlane T, Dionne MM, Trottier K, Olmsted MP. Development, feasibility, and acceptability of a brief, adjunctive cognitive-behavioral intervention aimed at encouraging rapid response to intensive eating disorder treatment. *Cogn Behav Pract*. 2021;28(1): 1–14. https://dx.doi.org/10.1016/j.cbpra.2020.05.007
84. Manasse SM, Lampe EW, Gillikin L, Payne-Reichert A, Zhang F, Juarascio AS, Forman EM. The project REBOOT protocol: Evaluating a personalized inhibitory control training as an adjunct to cognitive behavioral therapy for bulimia nervosa and binge-eating disorder. The *Int J Eating Disord*. 2020;53(6): 1007–1013. https://dx.doi.org/10.1002/eat.23225
85. Masheb RM, Grilo CM, Rolls BJ. A randomized controlled trial for obesity and binge eating disorder: Low-energy-density dietary counseling and cognitive-behavioral therapy. *Behav Res Ther*. 2011;49(12): 821–829. https://dx.doi.org/10.1016/j.brat.2011.09.006
86. McCabe M, Price E. Internet-based psychological and oral medical treatment compared to psychological treatment alone for ED. *J Sex Med*. 2008;5(10): 2338–2346. https://dx.doi.org/10.1111/j.1743-6109.2008.00885.x
87. McIntosh VVW, Jordan J, Carter JD, Frampton CMA, McKenzie JM, Latner JD, Joyce PR. Psychotherapy for transdiagnostic binge eating: A randomized controlled trial of cognitive-behavioural therapy, appetite-focused cognitive-behavioural therapy, and schema therapy. *Psychiatry Res*. 2016;240(qc4, 7911385): 412–420. https://dx.doi.org/10.1016/j.psychres.2016.04.080
88. Mental Health Services in the Capital Region, Denmark. *Training intervention in the treatment of anorexia nervosa*. Identifier NCT04185727. U.S. National Library of Medicine; 2019. https://classic.clinicaltrials.gov/show/NCT04185727.
89. Mills JS, Poulin LE, Kirsh G. Comparison of MI-oriented versus CBT-oriented adjunctive treatments: Impacts on therapeutic alliance and patient engagement during hospital treatment for an eating disorder. *Eating Disord*. 2023;11(1): 98. https://dx.doi.org/10.1186/s40337-023-00818-8
90. Mitchell JE, Pyle RL, Eckert ED, Hatsukami D, Pomeroy C, Zimmerman R. A comparison study of antidepressants and structured intensive group psychotherapy in the treatment of bulimia nervosa. *Arch Gen Psychiatry*. 1990;47(2): 149–157. https://dx.doi.org/10.1001/archpsyc.1990.01810140049008
91. Molinari E, Baruffi M, Croci M, Marchi S, Petroni ML. Binge eating disorder in obesity: Comparison of different therapeutic strategies. *Eat Weight Disord*. 2005;10(3): 154–161. https://dx.doi.org/10.1007/BF03327542
92. Naab S, Schlegl S, Korte A, Heuser J, Fumi M, Fichter M, et al. Effectiveness of a multimodal inpatient treatment for adolescents with anorexia nervosa in comparison with adults: An analysis of a specialized inpatient setting: Treatment of adolescent and adult anorexics. *Eat Weight Disord*. 2013;18(2): 167–173. https://dx.doi.org/10.1007/s40519-013-0029-8
93. Nagoya City University. *The remote family support programs for eating disorders*. Identifier NCT05840614. U.S. National Library of Medicine; 2023. https://classic.clinicaltrials.gov/show/NCT05840614.
94. Neumayr C, Voderholzer U, Tregarthen J, Schlegl S. Improving aftercare with technology for anorexia nervosa after intensive inpatient treatment: A pilot randomized controlled trial with a therapist-guided smartphone app. *Int J Eating Disord*. 2019;52(10): 1191–1201. https://dx.doi.org/10.1002/eat.23152
95. Newbridge House. *Evaluation of practical body image therapy for anorexia nervosa V1*. Identifier NCT04064255. U.S. National Library of Medicine; 2016. https://classic.clinicaltrials.gov/show/NCT04064255.
96. Newbridge House. *Evaluation of self-esteem group therapy for eating disorders*. Identifier NCT04072510. U.S. National Library of Medicine; 2018. https://classic.clinicaltrials.gov/show/NCT04072510.
97. Newbridge House. *Evaluation of compulsive exercise group therapy for eating disorders*. Identifier NCT04552639. U.S. National Library of Medicine; 2020. https://classic.clinicaltrials.gov/show/NCT04552639.
98. Nova Scotia Health Authority. *Effectiveness of the ECHOs approach for patients with eating disorders and their carers*. Identifier NCT01927042. U.S. National Library of Medicine; 2014. https://classic.clinicaltrials.gov/show/NCT01927042.
99. Nyman-Carlsson E, Norring C, Engstrom I, Gustafsson SA, Lindberg K, Paulson-Karlsson G, Nevonen L. Individual cognitive behavioral therapy and combined family/individual therapy for young adults with anorexia nervosa: A randomized controlled trial. *Psychother Res*. 2020;30(8): 1011–1025. https://dx.doi.org/10.1080/10503307.2019.1686190
100. Palmer RL, Birchall H, McGrain L, Sullivan V. Self-help for bulimic disorders: A randomised controlled trial comparing minimal guidance with face-to-face or telephone guidance. *Br J Psychiatry*. 2002;181(0342367, b1k): 230–235. https://dx.doi.org/10.1192/bjp.181.3.230
101. Pauli D, Flutsch N, Hilti N, Schraer C, Soumana M, Haberling I, Berger G. Home treatment as an add-on to family-based treatment in adolescents with anorexia nervosa: A pilot study. *Eur Eating Disord Rev*. 2022;30(2): 168–177. https://dx.doi.org/10.1002/erv.2882
102. Porras-Garcia B, Ferrer-Garcia M, Serrano-Troncoso E, Carulla-Roig M, Soto-Usera P, Miquel-Nabau H, et al. AN-VR-BE. A randomized controlled trial for reducing fear of gaining weight and other eating disorder symptoms in anorexia nervosa through virtual reality-based body exposure. *J Clin Med*. 2021;10(4): 682. https://dx.doi.org/10.3390/jcm10040682
103. Psychiatric University Hospital, Zurich*. Evaluation of the effectiveness of add-on hometreatment to family based therapy in adolescent anorexia nervosa*. Identifier NCT05418075. U.S. National Library of Medicine; 2022. https://classic.clinicaltrials.gov/show/NCT05418075.
104. Recovery Record Research. *Optimizing a smartphone application for individuals with eating disorders*. Identifier NCT02503098. U.S. National Library of Medicine; 2016. https://classic.clinicaltrials.gov/show/NCT02503098.
105. Reyes-Rodriguez ML. *Engaging Latino families in eating disorders treatment*. Identifier NCT01470508. U.S. National Library of Medicine; 2010. https://classic.clinicaltrials.gov/show/NCT01470508.
106. Rhodes P, Baillee A, Brown J, Madden S. Can parent-to-parent consultation improve the effectiveness of the Maudsley model of family-based treatment for anorexia nervosa? A randomized control trial. *J Fam Ther*. 2008;30(1): 96–108. https://dx.doi.org/10.1111/j.1467-6427.2008.00418.x
107. Riva G, Bacchetta M, Cesa G, Conti S, Molinari E. Six-month follow-up of in-patient experiential cognitive therapy for binge eating disorders. *Cyberpsychol Behav*. 2003;6(3): 251–258. https://dx.doi.org/10.1089/109493103322011533
108. Ruggeri M. *CBT–Eb plus EMDR versus CBT–Eb in patients with eating disorders*. Identifier NCT03156959. U.S. National Library of Medicine; 2017. https://classic.clinicaltrials.gov/show/NCT03156959.
109. Shalvata Mental Health Center. *Mindfulness training effect on self-monitoring adherence and group CBT outcomes for binge eating disorders*. Identifier NCT03094000. U.S. National Library of Medicine; 2015. https://classic.clinicaltrials.gov/show/NCT03094000.
110. Shalvata Mental Health Center. *The moderating role of baseline Oxytocin on its psychotherapy-facilitating effects among patients with eating disorders*. Identifier NCT05865288. U.S. National Library of Medicine; 2023. https://classic.clinicaltrials.gov/show/NCT05865288.
111. Shanghai Mental Health Center. *The effect of G-DBT on the patients with BN: A multicenter randomized controlled study*. Identifier NCT03455088. U.S. National Library of Medicine; 2018. https://classic.clinicaltrials.gov/show/NCT03455088.
112. Sheba Medical Center. *Cognitive behavioral therapy via a smartphone application on bulimia nervosa and binge eating disorder symptoms*. Identifier NCT02130037. U.S. National Library of Medicine; 2014. https://classic.clinicaltrials.gov/show/NCT02130037.
113. Shingleton RM, Pratt EM, Gorman B, Barlow DH, Palfai TP, Thompson-Brenner H. Motivational text message intervention for eating disorders: A single-case alternating treatment design using ecological momentary assessment. *Behav Ther*. 2016;47(3): 325–338. https://dx.doi.org/10.1016/j.beth.2016.01.005
114. St. Joseph's Healthcare Hamilton. *Using visual feedback to influence rapid response in the treatment of eating disorders*. Identifier NCT02940613. U.S. National Library of Medicine; 2016. https://classic.clinicaltrials.gov/show/NCT02940613.
115. Stanford University. *Adding guided self–help group therapy to the Alli Weight Loss Program in treating binge eating disorder*. Identifier NCT00601354. U.S. National Library of Medicine; 2007. https://classic.clinicaltrials.gov/show/NCT00601354.
116. Stanford University. *Effectiveness of cognitive remediation therapy in improving treatment retention in people with anorexia nervosa*. Identifier NCT00601822. U.S. National Library of Medicine; 2008. https://classic.clinicaltrials.gov/show/NCT00601822.
117. Steinglass JE, Albano AM, Simpson HB, Wang Y, Zou J, Attia E, Walsh BT. Confronting fear using exposure and response prevention for anorexia nervosa: A randomized controlled pilot study. *Int J Eating Disord*. 2014;47(2): 174–180. https://dx.doi.org/10.1002/eat.22214
118. Tanaka M, Nakahara T, Muranaga T, Kojima S, Yasuhara D, Ueno H, et al. Ghrelin concentrations and cardiac vagal tone are decreased after pharmacologic and cognitive-behavioral treatment in patients with bulimia nervosa. *Horm Behav*. 2006;50(2): 261–265. https://dx.doi.org/10.1016/j.yhbeh.2006.03.009
119. Thompson-Brenner H, Boswell JF, Espel-Huynh H, Brooks G, Lowe MR. Implementation of transdiagnostic treatment for emotional disorders in residential eating disorder programs: A preliminary pre-post evaluation. *Psychot Res*. 2019;29(8): 1045–1061. https://dx.doi.org/10.1080/10503307.2018.1446563
120. Timko CA, Bhattacharya A, Fitzpatrick KK, Howe H, Rodriguez D, Mears C, et al. The shifting perspectives study protocol: Cognitive remediation therapy as an adjunctive treatment to family based treatment for adolescents with anorexia nervosa. *Contemp Clin Trials*. 2021;103(101242342): 106313. https://dx.doi.org/10.1016/j.cct.2021.106313
121. Treasure JL, Katzman M, Schmidt U, Troop N, Todd G, de Silva P. Engagement and outcome in the treatment of bulimia nervosa: First phase of a sequential design comparing motivation enhancement therapy and cognitive behavioural therapy. *Behav Res Ther*. 1999;37(5): 405–418. https://dx.doi.org/10.1016/S0005-7967(98)00149-1
122. Tronieri JS, Wadden TA, Walsh O, Berkowitz RI, Alamuddin N, Gruber K, et al. Effects of liraglutide on appetite, food preoccupation, and food liking: Results of a randomized controlled trial. *Int J Obes*. 2020;44(2): 353–361. https://dx.doi.org/10.1038/s41366-019-0348-6
123. Universitaire Ziekenhuizen KU Leuven. *Dopamine release to food reward in bulimia nervosa*. Identifier NCT03471806. U.S. National Library of Medicine; 2018. https://classic.clinicaltrials.gov/show/NCT03471806.
124. University Health Network, Toronto. *Integrated treatment for co-occurring eating disorders and posttraumatic stress disorder*. Identifier NCT03502564. U.S. National Library of Medicine; 2015. https://classic.clinicaltrials.gov/show/NCT03502564.
125. University of Barcelona. *Virtual reality based cue-exposure treatment for bulimia nervosa*. Identifier NCT02237300. U.S. National Library of Medicine; 2015. https://classic.clinicaltrials.gov/show/NCT02237300.
126. University of Barcelona. *Virtual reality-based attention bias modification training for anorexia nervosa (AN-VR-ABM)*. Identifier NCT04786951. U.S. National Library of Medicine; 2021. https://classic.clinicaltrials.gov/show/NCT04786951.
127. University of Barcelona. *Improving the treatment of anorexia nervosa in children through virtual reality body exposure*. Identifier NCT06166355. U.S. National Library of Medicine; 2024. https://classic.clinicaltrials.gov/show/NCT06166355.
128. University of California, San Diego. *Binge eating anxiety and mood*. Identifier NCT04242550. U.S. National Library of Medicine; 2020. https://classic.clinicaltrials.gov/show/NCT04242550.
129. University of Edinburgh. Eating Disorders. *Online self-help & usual treatment (TAU) vs TAU only*. Identifier NCT03107221. U.S. National Library of Medicine; 2016. https://classic.clinicaltrials.gov/show/NCT03107221.
130. University of North Carolina, Chapel Hill. *UCAN2:* *Uniting couples in the treatment of anorexia nervosa*. Identifier NCT01740752. U.S. National Library of Medicine; 2012. https://classic.clinicaltrials.gov/show/NCT01740752.
131. University of North Carolina, Charlotte. *Empowering mindfulness, body respect, and compassionate eating among women who binge eat during pregnancy*. Identifier NCT05581095. U.S. National Library of Medicine; 2023. https://classic.clinicaltrials.gov/show/NCT05581095.
132. University of Turin, Italy. *Neurobiology of eating disorders treatments*. Identifier NCT01990755. U.S. National Library of Medicine; 2010. https://classic.clinicaltrials.gov/show/NCT01990755.
133. VA Office of Research and Development. *Weight loss treatment for veterans with binge eating*. Identifier NCT03234881. U.S. National Library of Medicine; 2018. https://classic.clinicaltrials.gov/show/NCT03234881.
134. Van Passel B, Danner UN, Dingemans AE, Aarts E, Sternheim LC, Becker ES, et al. Cognitive remediation therapy does not enhance treatment effect in obsessive-compulsive disorder and anorexia nervosa: A randomized controlled trial. *Psychother Psychosom*. 2020;89(4): 228–241. https://dx.doi.org/10.1159/000505733
135. Vander Wal JS, Maraldo TM, Vercellone AC, Gagne DA. Education, progressive muscle relaxation therapy, and exercise for the treatment of night eating syndrome. A pilot study. *Appetite*. 2015;89(6jw, 8006808): 136–144. https://dx.doi.org/10.1016/j.appet.2015.01.024
136. Vandereycken W, Pierloot R. Pimozide combined with behavior therapy in the short-term treatment of anorexia nervosa. A double-blind placebo-controlled cross-over study. *Acta Psychiatr Scand*. 1982;66(6): 445–450. https://doi.org/10.1111/j.1600-0447.1982.tb04501.x
137. Ventura M, Bauer B. Empowerment of women with purging-type bulimia nervosa through nutritional rehabilitation. *Eat Weight Disord*. 1999;4(2): 55–62. https://dx.doi.org/10.1007/BF03339719
138. Walsh BT, Wilson GT, Loeb KL, Devlin MJ, Pike KM, Roose SP, et al. Medication and psychotherapy in the treatment of bulimia nervosa. *Am J Psychiatry*. 1997;154(4): 523–531.
139. Walsh BT, Fairburn CG, Mickley D, Sysko R, Parides MK. Treatment of bulimia nervosa in a primary care setting. *Am J Psychiatry*. 2004;161(3): 556–561. https://doi.org/10.1176/appi.ajp.161.3.556
140. Wang C, Xiao R. Music and art therapy combined with cognitive behavioral therapy to treat adolescent anorexia patients. *Am J Transl Res*. 2021;13(6): 6534–6542.
141. Washington University School of Medicine. *Helping HAND: Healing anorexia nervosa digitally*. Identifier NCT05499676. U.S. National Library of Medicine; 2022. https://classic.clinicaltrials.gov/show/NCT05499676.
142. Wilks D. *The role of emotional dysregulation in the treatment of eating disorders*. PhD thesis. North Central University; 2006.
143. Wnuk S. *Treatment development and evaluation of emotion-focused group therapy for women with symptoms of bulimia nervosa*. PhD thesis. York University; 2011.
144. Woolhouse H, Knowles A, Crafti N. Adding mindfulness to CBT programs for binge eating: A mixed-methods evaluation. *Eating Disord*. 2012;20(4): 321–339. https://dx.doi.org/10.1080/10640266.2012.691791
145. Yale University. *Behavioral and pharmacologic treatment of binge eating and obesity: Specialist treatment*. Identifier NCT03063606. U.S. National Library of Medicine; 2017. https://classic.clinicaltrials.gov/show/NCT03063606.
